# Evaluation of the Clinical and Microbiological Response to *Salmonella* Paratyphi A Infection in the First Paratyphoid Human Challenge Model

**DOI:** 10.1093/cid/cix042

**Published:** 2017-02-04

**Authors:** Hazel C. Dobinson, Malick M. Gibani, Claire Jones, Helena B. Thomaides-Brears, Merryn Voysey, Thomas C. Darton, Claire S. Waddington, Danielle Campbell, Iain Milligan, Liqing Zhou, Sonu Shrestha, Simon A. Kerridge, Anna Peters, Zoe Stevens, Audino Podda, Laura B. Martin, Flavia D’Alessio, Duy Pham Thanh, Buddha Basnyat, Stephen Baker, Brian Angus, Myron M. Levine, Christoph J. Blohmke, Andrew J. Pollard

**Affiliations:** 1 Oxford Vaccine Group, Department of Paediatrics, University of Oxford, and the National Institute for Health Research Oxford Biomedical Research Centre, and; 2 Nuffield Department of Primary Care Health Sciences, University of Oxford, United Kingdom;; 3 GSK Vaccines Institute for Global Health, Siena, Italy;; 4 European Vaccine Initiative, Heidelberg, Germany;; 5 Hospital for Tropical Diseases, Wellcome Trust Major Overseas Programme, Oxford University Clinical Research Unit, Ho Chi Minh City, Vietnam;; 6 Oxford University Clinical Research Unit, Patan Academy of Health Sciences, Kathmandu, Nepal;; 7 Centre for Tropical Medicine and Global Health, University of Oxford,; 8 London School of Hygiene and Tropical Medicine, and; 9 Nuffield Department of Medicine, University of Oxford, United Kingdom; and; 10 Center for Vaccine Development, University of Maryland School of Medicine, Baltimore

**Keywords:** paratyphoid infection, enteric fever, *Salmonella enterica* paratyphi A, human challenge study, immune responses

## Abstract

**Background.:**

To expedite the evaluation of vaccines against paratyphoid fever, we aimed to develop the first human challenge model of *Salmonella enterica* serovar Paratyphi A infection.

**Methods.:**

Two groups of 20 participants underwent oral challenge with *S*. Paratyphi A following sodium bicarbonate pretreatment at 1 of 2 dose levels (group 1: 1–5 × 10^3^ colony-forming units [CFU] and group 2: 0.5–1 × 10^3^ CFU). Participants were monitored in an outpatient setting with daily clinical review and collection of blood and stool cultures. Antibiotic treatment was started when prespecified diagnostic criteria were met (temperature ≥38°C for ≥12 hours and/or bacteremia) or at day 14 postchallenge.

**Results.:**

The primary study objective was achieved following challenge with 1–5 × 10^3^ CFU (group 1), which resulted in an attack rate of 12 of 20 (60%). Compared with typhoid challenge, paratyphoid was notable for high rates of subclinical bacteremia (at this dose, 11/20 [55%]). Despite limited symptoms, bacteremia persisted for up to 96 hours after antibiotic treatment (median duration of bacteremia, 53 hours [interquartile range, 24–85 hours]). Shedding of *S*. Paratyphi A in stool typically preceded onset of bacteremia.

**Conclusions.:**

Challenge with *S.* Paratyphi A at a dose of 1–5 × 10^3^ CFU was well tolerated and associated with an acceptable safety profile. The frequency and persistence of bacteremia in the absence of clinical symptoms was notable, and markedly different from that seen in previous typhoid challenge studies. We conclude that the paratyphoid challenge model is suitable for the assessment of vaccine efficacy using endpoints that include bacteremia and/or symptomatology.

**Clinical Trials Registration.:**

NCT02100397.

## Summary

The safe establishment of a protocol for a human challenge model for *Salmonella* Paratyphi A can be used to expedite the evaluation of novel vaccine candidates and provides insight into the clinical and immune response to paratyphoid infection.

Efforts aimed at reducing the global burden of enteric fever are likely to require a coordinated strategy comprising improvements in water quality, sanitation, and hygiene measures along with the development of effective vaccines. While *Salmonella enterica* serovar Typhi remains the principal etiological agent of enteric fever globally, *Salmonella enterica* serovar Paratyphi A (*S.* Paratyphi A) is responsible for an increasing proportion of enteric fever cases. In South and Southeast Asia, annual incidence rates in some areas are estimated to be as high as 150 cases per 100000 person-years [1].

Relatively little is known regarding the pathophysiology and host response to *S.* Paratyphi A infection. *Salmonella* Typhi and Paratyphi A possess similar genomic markers of host restriction, but differ with regards to expression of certain virulence factors, such as the lack of Vi polysaccharide capsule expression by *S.* Paratyphi A/B [2, 3]. Additionally, there are currently no licensed vaccines available for the prevention of paratyphoid fever. The licensed *S.* Typhi vaccine Ty21a can induce cross-reactive humoral immune responses to *S.* Paratyphi A/B in vitro, and there is some evidence from field trials for cross-protection against *S.* Paratyphi B [4, 5]. Several promising vaccine candidates are in development, including live-attenuated strains and conjugate vaccines targeted against the O-specific polysaccharide (O:2) of *S.* Paratyphi A, although no candidate vaccines have undergone efficacy trials to date [6]. Development of vaccines to prevent paratyphoid infection is hampered by limited knowledge of immunological correlates of protection and the lack of a suitable small-animal model of infection.

Human challenge studies are increasingly used to identify promising vaccine candidates suitable for evaluation in large-scale field trials [7]. In this study, we sought to establish an *S.* Paratyphi A human challenge model in healthy adult volunteers by oral challenge to enable future assessment of *S.* Paratyphi A vaccines and to assess host–pathogen interactions under a strictly controlled setting.

## METHODS

### Study Design

We performed an observational, dose-level modification human challenge study of *S.* Paratyphi A infection conducted in healthy community adult volunteers, at the Centre for Clinical Vaccinology and Tropical Medicine, University of Oxford, United Kingdom [8]. Description of the study protocol and enrollment criteria are detailed elsewhere [9, 10].

The primary objective of the study was to determine the dose in colony-forming units (CFU) of *S.* Paratyphi A strain NVGH308 required to achieve an attack rate of 60%–75%, when ingested with sodium bicarbonate solution to neutralize gastric acid.

### Challenge Strain

*Salmonella* Paratyphi A strain NVGH308 was isolated in 2006 from a patient with paratyphoid fever in Kathmandu, Nepal, and is susceptible to most commonly used antibiotics, including ciprofloxacin (Supplementary Table 1) [9]. The NVGH308 strain was selected as it is a contemporary, circulating strain isolated from a patient in a highly endemic country.

Whole-genome sequencing was undertaken to identify the phylogenetic relationship of NVGH308 to other circulating strains (Supplementary Figure 1; Supplementary Methods).

The challenge inoculum was freshly prepared on day of challenge (Supplementary Methods). An initial target dose of 1–5 × 10^3^ CFU (group 1) was selected based upon prior *S.* Typhi challenge studies in a similar study population [11]. Following fulfillment of the primary objective at the initial target dose, we amended the original study protocol to include a dose de- escalation group (0.5–1 × 10^3^ CFU; group 2) to affirm a relationship between dose and diagnostic endpoints.

### Clinical Evaluation

Regular participant safety monitoring occurred through daily clinical review for a minimum of 14 days and included solicited symptoms (Supplementary Table 2) and twice-daily temperature measurements [9].

### Diagnostic Criteria

The primary outcome was the rate of paratyphoid infection, defined as a positive blood culture for *S.* Paratyphi A (taken ≥72 hours after challenge to avoid detection of primary bacteremia) and/or oral temperature ≥38°C persisting for ≥12 hours [9]. Treatment was initiated upon fulfillment of diagnostic criteria (or at day 14 for those without illness) and comprised oral ciprofloxacin 500 mg twice daily for 14 days.

### Serological Response

Blood was collected at baseline and days 10, 28, and 90 post-challenge. Specific immunoglobulin M (IgM), immunoglobulin G (IgG), and immunoglobulin A (IgA) to *S.* Paratyphi A lipopolysaccharide (LPS) and flagellin (H) were measured by an in-house enzyme-linked immunosorbent assay using goat antihuman IgM, IgG, and IgA conjugated to horseradish peroxidase (Supplementary Methods).

### Statistical Analysis

All participants were included in the primary analysis and a post hoc analysis was conducted comparing participants challenged with *S*. Paratyphi A in this study with those challenged with *S.* Typhi in our previous study [11].

Clinical, laboratory, and immunological data were collated using a clinical trials database (OpenClinica, version 3.1). Data analysis was performed as described in detail in the Supplementary Methods, using R version 3.2.2 [12].

## RESULTS

Forty healthy adult participants were enrolled into the study between 20 May and 20 November 2014 (Table 1; Supplementary Figure 2). Twenty participants were challenged with *S.* Paratyphi A at a target dose of 1–5 × 10^3^ CFU (group 1). A second group of 20 participants was challenged at a target dose of 0.5–1.0 × 10^3^ CFU (group 2).

**Table 1.** Participant Demographic Characteristics

Characteristic	Group 1	Group 2	All
No. of participants challenged	20	20	40
Sex, male	10 (50)	11 (55)	21 (52.5)
Age, y, median (range)	27 (19–49)	23 (20–50)	25 (19–50)
Ethnicity, white British	18 (90)	17 (85)	35 (88)
Country of birth, United Kingdom	15 (75)	17 (85)	32 (80)
Tobacco smoking ever, yes	9 (45)	8 (40)	17 (43)
Alcohol intake, units/wk, median (IQR)	4 (0–10)	7 (4–11)	5 (2–10)
BMI, kg/m^2^, mean ± SD	23.5 ± 3.2	22.8 ± 4.2	24.1 ± 3.8

Data are presented as No. (%) unless otherwise indicated.

Abbreviations: BMI, body mass index; IQR, interquartile range; SD, standard deviation.

**Figure 1.**
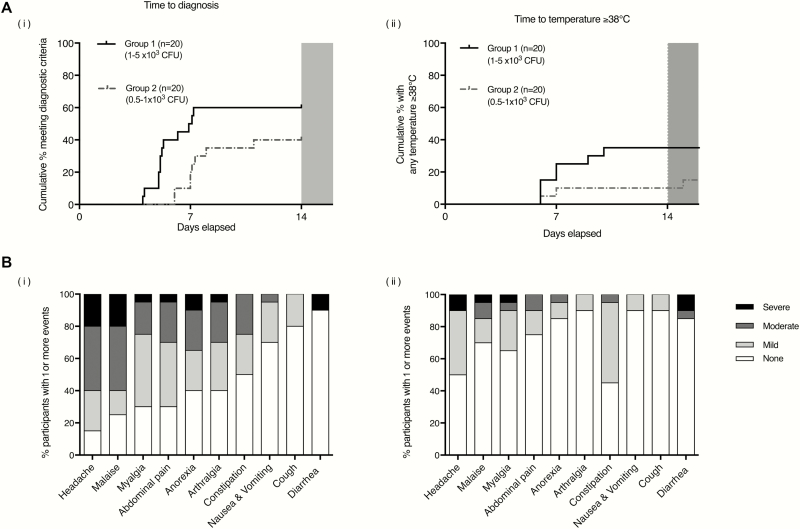
*A*, Kaplan-Meier plots indicating time to diagnostic endpoints: time to diagnosis (*i*) and time to first temperature ≥38°C (*ii*). *B*, Clinical symptom profiles following *Salmonella* Paratyphi A challenge. Percentage of participants reporting solicited systemic symptoms on 1 or more days following *S*. Paratyphi A challenge, recorded using an online diary for 21 days. Stacked columns display percentage of participants reporting maximum symptom severity as mild, moderate, or severe. (*i*) Individuals meeting prespecified criteria for paratyphoid disease in group 1 and group 2. (*ii*) Individuals who did not meet prespecified criteria for paratyphoid disease in group 1 and group 2. None, no reported symptoms; mild, present but no limitation of usual activity; moderate, some limitation of daily activity; severe, unable to perform normal daily activity. Abbreviation: CFU, colony-forming units.

Paratyphoid infection was diagnosed in 50% (20/40) of all participants challenged. The primary study objective was achieved in challenge group 1, where 12 of 20 participants met the prespecified diagnostic criteria for paratyphoid fever (attack rate, 60%; 95% confidence interval [CI], 36%–81%) (Figure 1). Eleven participants (11/12 [92%]) in this group were diagnosed following a positive blood culture for *S.* Paratyphi A and 1 participant was diagnosed based on temperature criteria with subsequent blood culture confirmation. Median time from challenge to initiation of treatment was 6.4 days (range, 5.9–8.3 days) (Table 2).

**Table 2.** Attack Rates and Response to *Salmonella* Paratyphi A Challenge

Results	Group 1 (n = 20)	Group 2 (n = 20)	All (N = 40)
Target challenge dose, CFU	1–5 × 10^3^	0.5–1 × 10^3^	…
Attack rate, No. (% total) (95% CI)	12 (60) (36–81)	8 (40) (19–64)	20 (50) (34–66)
Actual challenge dose, CFU × 10^3^, median (range)	2.4 (2.2–2.8)	0.9 (0.7–1.3)	…
Paratyphoid A diagnosed	2.4 (2.2–2.7)	1.0 (0.7–1.3)	…
Paratyphoid A not diagnosed	2.5 (2.3–2.7)	0.8 (0.7–1.3)	…
Actual challenge dose/body surface area^a^, CFU/m^2^, mean ± SD	1350 ± 166	519 ± 163	…
Paratyphoid A diagnosed	1336 ± 196	533 ± 171	…
Paratyphoid A not diagnosed	1371 ± 119	509 ± 165	…
Mode of diagnosis, No. (% total)			
Temperature ≥38°C for ≥12 h with blood culture confirmation	1 (5)	0 (0)	1 (2.5)
Microbiological diagnosis^b^	11 (55)	8 (40)	19 (47.5)
Bacteraemia, No. (% total)	12 (60)	8 (40)	20 (50)
Time to bacteremia^c^, d, median (IQR)	5.2 (5.0–6.4)	7 (6–11)	6.1 (5.1–7.1)
Time to antibiotics^d^, d, median (IQR)	6.4 (6.2–7.4)	8.3 (8.2–9.2)	8.0 (6.4–8.3)
Overall duration of bacteremia^e^, d, median (IQR)	4.1 (3.4–5.0)	1.0 (1.0 -3.4)	4.0 (1.1–4.9)
Clearance time^f^, d, median (IQR)	2.9 (2.1–3.6)	0.9 (0.7–1.9)	2.2 (1.0–3.5)
Quantitative blood culture, CFU/mL, median (range)	1.1 (0–4.1)	0.2 (0–6.9)	1 (0–6.9)
Temperature ≥38°C (any duration)^g^, No. (% total)	7 (35)	2 (10)	9 (45)
Paratyphoid A diagnosed	7 (35)	2 (10)	9 (45)
Paratyphoid A not diagnosed	0 (0)	0 (0)	0 (0)
Stool shedding, No. (% total)	12 (60)	4 (20)	16 (40)
Paratyphoid A diagnosed	8 (40)	2 (10)	10 (25)
Paratyphoid A not diagnosed	4 (20)	2 (10)	6 (15)
Attack rates using alternative diagnostic criteria			
Temperature ≥37.5°C (any duration), No. (% total)	8 (40)	4 (20)	12 (30)
Temperature ≥38°C (any duration), No. (% total)	7 (35)	2 (10)	9 (45)
Temperature ≥37.5°C (any duration) and positive blood culture	7 (35)	4 (20)	11 (27.5)
Temperature ≥38°C (any duration) and positive blood culture	7 (35)	2 (10)	9 (22.5)

Abbreviations: CFU, colony-forming units; CI, confidence interval; IQR, interquartile range; SD, standard deviation.

^a^Body surface area (BSA) calculated according to Mosteller method: BSA (m^2^) = (height in cm × weight in kg / 3600) × 0.5.

^b^ *Salmonella* Paratyphi A isolated from blood culture taken ≥72 h from challenge.

^c^Time from challenge to collection of first positive blood culture.

^d^Time from ingestion of the challenge agent to the fulfillment of diagnostic criteria.

^e^Time from collection of first positive blood culture to collection of first persistently negative blood culture.

^f^Time from initiation of antibiotics to collection of first persistently negative blood culture.

^g^Day 0 (day of challenge) to day 14.

Paratyphoid infection was diagnosed less frequently in challenge group 2. In this group, 8 of 20 participants met the prespecified diagnostic criteria following challenge (attack rate, 40%; 95% CI, 19%–64%), all of whom were diagnosed following a positive blood culture (Table 2). Median time from challenge to initiation of treatment was 8.3 days (range, 7.1–12.0 days) (Figure 1).

Attack rates using alternative diagnostic criteria are outlined in Table 2. The hypothetical attack rate was lower in both study groups if clinical endpoints alone (eg, fever) were used to define paratyphoid disease.

All participants were managed with daily clinical review and no participants met the prespecified criteria for severe enteric fever [9]. Two participants had positive stool cultures for *S.* Paratyphi A after completing a 14-day course of ciprofloxacin, neither of whom had evidence of gallbladder disease at screening. Both were successfully re-treated with a 14-day course of oral azithromycin (500 mg daily), and all subsequent clearance stools were negative. There were no episodes of relapse of clinical disease [9]. One serious adverse event was recorded (appendicitis 6 months after *S.* Paratyphi A challenge) and was assessed as being unrelated to study procedures. One participant developed mouth ulcers after treatment with ciprofloxacin, which resolved following a change to azithromycin. A second participant developed vaginal candidiasis following antibiotic treatment (ciprofloxacin), which resolved following treatment with topical clotrimazole.

**Figure 2.**
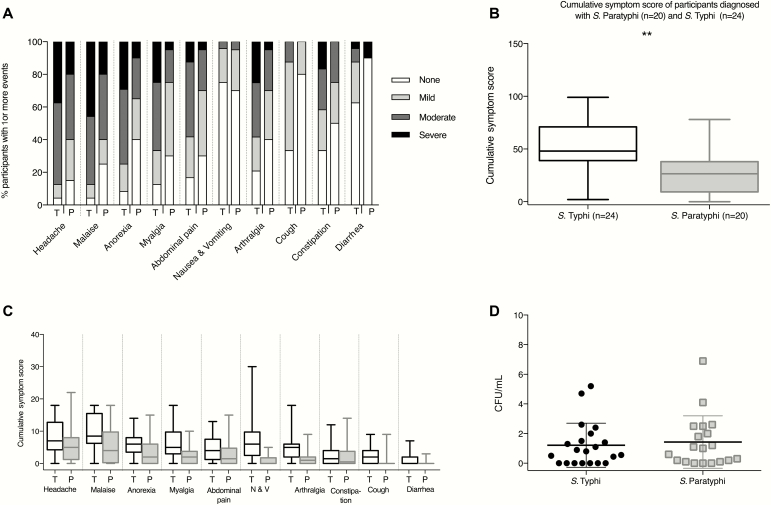
Comparison of the clinical and microbiological response to *Salmonella* Typhi and *Salmonella* Paratyphi A challenge. *A*, Solicited systemic symptoms in individuals with acute typhoid (n = 24) or paratyphoid (n = 20) disease recorded using an online diary (day 0, day of challenge; day 14, final day of treatment initiation). Percentage of participants reporting 1 or more events. Stacked columns display percentage of participants reporting maximum symptom severity as mild (present but no limitation of usual activity), moderate (some limitation of daily activity), or severe (unable to perform normal daily activity). *B*, Comparison of cumulative symptom severity scores between participants with acute typhoid (n = 24) or paratyphoid (n = 20) disease. Symptom scores were calculated by summing numerical values assigned to the severity of individual symptoms between day 0 and day 14 (0, not present; 1, mild; 2, moderate; 3, severe). Box-and-whisker plots display median, interquartile range, and range. *C*, Symptom severity scores for specific symptoms, day 0 to day 14. *D*, Results of quantitative blood cultures collected immediately prior to initiation of antibiotic treatment for participants with typhoid or paratyphoid disease (see Materials and Methods). Abbreviations: CFU, colony-forming units; N & V, nausea and vomiting; P, paratyphoid; T, typhoid.

The most common symptom reported by those with paratyphoid illness was headache (17/20 [85%]), followed by malaise (15/20 [75%]), abdominal pain (14/20 [70%]), and myalgia (14/20 [70%]). The majority of solicited symptoms were graded as mild and did not result in limitation of usual activity (Figure 1; Supplementary Table 2). In comparison with participants challenged with *S.* Typhi in earlier studies [11], paratyphoid resulted in a milder symptom profile, characterized by fewer severe symptoms and lower cumulative symptom scores (Figure 2; Supplementary Table 3).

Laboratory parameters were in keeping with those expected for acute enteric fever (Supplementary Figure 3) [13]. Among those meeting diagnostic endpoints, peripheral blood biochemistry demonstrated elevated C-reactive protein and alanine aminotransferase, while hematology revealed a modest fall in hemoglobin, neutrophil, and lymphocyte counts.

**Figure 3.**
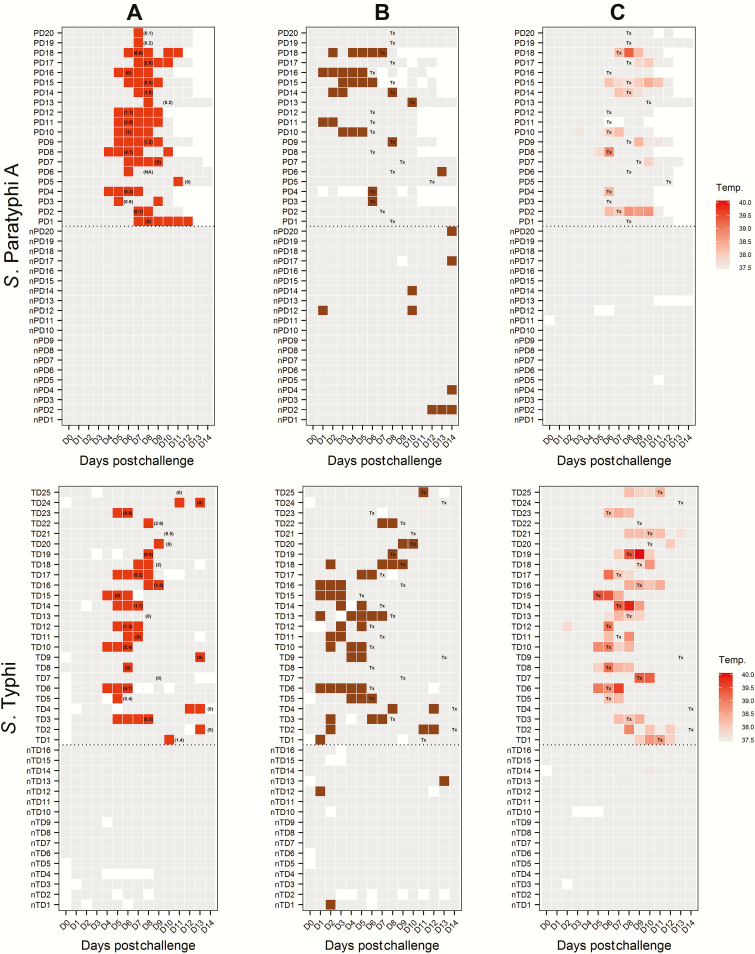
Clinical and microbiological dynamics for participants challenged with *Salmonella* Typhi and *Salmonella* Paratyphi A. *A*, Pattern of blood culture results (red, positive; gray, negative; white, sample not collected). Numbers in parentheses refer to quantitative blood culture colony count (colony-forming units/mL) taken immediately prior to antibiotic treatment and correspond to day of treatment initiation. *B*, Pattern of stool shedding following challenge. Colored squares indicate stool culture results for *S*. Paratyphi A or *S.* Typhi (brown, positive; gray, negative; white, sample not collected). *C*, Maximum temperature (temp., °C) measurements by day. Abbreviations: day 0, day of challenge; nPD1–20, paratyphoid disease not diagnosed; nTD1–16, typhoid disease not diagnosed; PD1–20, confirmed paratyphoid disease; TD1–25, confirmed typhoid disease; Tx, day of treatment initiation.

We documented at least 1 positive blood culture in all participants diagnosed with paratyphoid (20/20 [100%]; median number of positive blood cultures, 5.5; range, 1–8). Median bacteremia clearance times were 2.2 days (interquartile range [IQR], 1–3.5 days) for *S*. Paratyphi (Figures 2 and 3). The median time from challenge to bacteremia was 5.2 days (IQR, 5.0–6.4 days) in group 1 and 7 days (IQR, 6.7–7.4 days) in group 2. Quantitative blood cultures performed prior to antibiotic treatment demonstrated median bacterial loads of 1.1 CFU/mL (range, 0–4.1 CFU/mL) in group 1 and 0.2 CFU/mL (range, 0–6.9 CFU/mL) in group 2 participants, which are comparable to those seen in cases of typhoid fever in earlier challenge studies and in endemic settings [11, 14]. The rate of blood culture positive days was twice as high in *S*. Paratyphi A–diagnosed participants than in *S*. Typhi–diagnosed participants from our previous study (incidence rate ratio [IRR], 2.1; 95% CI, 1.46–3.1; *P* < .001) (Figure 2; Table 2).

Subclinical bacteremia was a common finding following challenge, with 11 of 20 (55%; 95% CI, 32%–77%) participants having demonstrable bacteremia but remaining persistently afebrile following challenge, although other symptoms were observed as discussed above (Supplementary Table 2). Afebrile bacteremia was less common in earlier typhoid challenge studies performed by our group (4/21 [19%]; 95% CI, 5%–42%; *P* = .025, Fisher exact test) [11]. Fever was recorded less frequently in group 2 (2/8 [25%]) than in group 1 (7/12 [58%]) (Table 2).

Shedding of *S.* Paratyphi A in stool occurred sporadically throughout the challenge period, and at least 1 stool culture was positive in 17 of 40 participants (43%; 95% CI, 27%–59%) (Figure 3). Among participants with demonstrable bacteremia, 11 of 20 (55%; 95% CI, 32%–77%) had a positive stool culture for at least 1 time point and stool shedding frequently preceded the onset of bacteremia (Figure 3). Asymptomatic shedding was identified in 6 of 20 participants (30%; 95% CI, 12%–54%) who did not reach the diagnostic criteria during the observation period. The rate of stool positivity was similar in *S*. Paratyphi A–diagnosed participants as was observed previously in *S*. Typhi–diagnosed participants (IRR, 0.78; 95% CI, .40–1.52; *P* = .457).

Baseline anti-LPS IgG geometric mean concentrations were higher in undiagnosed participants prior to challenge (*P* = .034) (Supplementary Figure 4). Anti-LPS antibody responses peaked at day 28 postchallenge and were significantly higher in those diagnosed with paratyphoid compared with those without acute disease (*P* < .001). In contrast, anti-H antibody responses were only marginally increased above baseline at D28 and only in those meeting diagnostic endpoints (Supplementary Figure 4).

## DISCUSSION

In 1909, Proescher reported a case of “two individuals who accidentally swallowed a small amount of a pure culture” of *Salmonella* Paratyphi A and who “became ill … five and seven days after imbibing” the culture [15]. Here, we describe the first deliberate and controlled human challenge model using a contemporary circulating strain of *S.* Paratyphi A. Although *S*. Typhi challenge studies have become well established over the past half-century, we have uniquely demonstrated that *S.* Paratyphi A challenge can also be undertaken safely in an outpatient setting. Challenge at a target dose of 1–5 × 10^3^ CFU was sufficient to achieve an attack rate of 60% in a cohort of naive volunteers, whereas challenge using a lower dose resulted in a reduced attack rate and increased time to bacteremia.

While Vi-conjugate typhoid vaccines are currently licensed in both India and China, the paratyphoid A vaccine pipeline is less well developed [6]. The effect of future Vi-based typhoid vaccine programs on the burden of paratyphoid fever is unknown and there is conflicting evidence of serovar replacement following historical typhoid vaccination campaigns [16, 17]. This presents a pressing need for accelerated paratyphoid A vaccine development, which could be facilitated by the establishment of a human paratyphoid A challenge model. In support of this, the World Health Organization has stated that well-validated challenge studies can provide “considerable supporting evidence of the efficacy” of typhoid Vi-conjugate vaccines, as well as offering insights into immunological correlates of protection [18]. We suggest that the model described herein could fulfill an equivalent role for paratyphoid by providing a platform to assess the efficacy of paratyphoid vaccines currently in development.

The choice of diagnostic endpoints is central to future applications of this model in the assessment of candidate paratyphoid vaccines. In this study, we used a composite diagnostic endpoint of clinical and/or microbiologically defined paratyphoid infection and observed that 95% of individuals who met the diagnostic criteria did so based upon microbiological criteria (Table 2). The frequency of blood culture sampling in this study resulted in the identification of several cases of asymptomatic bacteremia, which may or may not have progressed to symptomatic paratyphoid fever if left untreated. While the consistent finding of bacteremia represents an unambiguous diagnostic endpoint, this could set an overly stringent threshold in future vaccine efficacy studies, and clinical endpoints might be preferable as this more accurately reflects the situation in the field (Table 2). Arguably, vaccines demonstrating at least moderate efficacy in this challenge model would perform better in field trials, where the endpoints would include a clinical syndrome of paratyphoid fever with culture confirmation. Vaccine efficacy studies using this model should include multiple alternative prespecified secondary diagnostic endpoints, such as the proportion of cases diagnosed by clinical criteria alone or microbiological criteria alone or a composite of clinical symptoms with culture confirmation.

To our knowledge, no previous studies have explored the infectious dose required to induce paratyphoid disease, although it is frequently assumed that *S*. Paratyphi A requires a higher dose of bacteria to cause clinical disease than does *S*. Typhi [19, 20]. In this study, an attack rate of 60% was achieved using a dose of 1–5 × 10^3^ CFU *S*. Paratyphi A, which is comparable to the attack rate (55%) observed at an equivalent dose of *S*. Typhi in earlier studies [11]. Notably, challenge with as few as 700 CFU *S.* Paratyphi A was able to induce acute disease, in the context of sodium bicarbonate pretreatment. Our findings are broadly consistent with observations from *S*. Typhi challenge studies, where reducing the challenge inoculum results in a reduced attack rate and longer incubation period [21]. As with *S*. Typhi, it is possible that the infectious dose required to induce paratyphoid disease in a nonendemic setting may differ from that required in an endemic setting, where prior exposure could lead to partial immunity.

We performed a post hoc comparison of symptom data collected during earlier typhoid challenge studies [11] and noted that paratyphoid challenge resulted in a milder disease profile than that previously observed following typhoid challenge. In our model, paratyphoid was characterized by high rates of afebrile bacteremia in groups 1 and 2 (11/20 [55%]) with minimal symptoms, which may otherwise have escaped detection in the absence of intensive sampling. Case series from the preantibiotic era reported that *S.* Paratyphi A caused a spectrum of clinical manifestations that “closely resembles that of mild enteric fever” [22], and asymptomatic cases of paratyphoid infection are well described [23, 24]. Although the largest published case series to date indicates that *S.* Typhi and Paratyphi A infections cause an indistinguishable clinical syndrome [19], this study was limited to patients who attended healthcare facilities and may not account for ambulatory or mild cases of paratyphoid in the community. In keeping with this, epidemiological data from endemic settings suggest that subclinical paratyphoid infection frequently occurs [5]. In light of our challenge model data, we interpret earlier data as being supportive of the hypothesis of an underappreciated and undiagnosed burden of paratyphoid disease in the community. In a proportion of individuals, exposure to the bacillus is followed by asymptomatic bacteremia and asymptomatic stool shedding, potentially perpetuating both long- and short-cycle transmission. In keeping with this, asymptomatic shedding of *S*. Paratyphi A was also observed in our model, including in individuals who did not meet diagnostic criteria for paratyphoid disease.

Somewhat counterintuitively, we observed prolonged bacteremia and protracted time to blood culture clearance (≥96 hours) in the paratyphoid challenge model, despite the low rate of fever. In addition, convalescent stool shedding was noted in 2 individuals despite a 14-day course of ciprofloxacin, both of whom were successfully treated with a further course of azithromycin. The relatively poor response to ciprofloxacin was unexpected, given that the NVGH308 strain is sensitive to ciprofloxacin according to current Clinical and Laboratory Standards Institute and European Committee on Antimicrobial Susceptibility Testing guidelines (minimum inhibitory concentration [MIC] = 0.06 μg/mL) [25, 26]. Studies in endemic settings have found that fluoroquinolone MICs for *S*. Paratyphi A were typically higher than those for *S*. Typhi [19]. The mechanisms underlying the discrepancy between the clinical and microbiological profiles of paratyphoid infection seen in this study are unclear, but may represent a strategy of achieving infection-by-stealth that facilitates onward transmission and persistence of the bacterium within the environment.

Ethical and safety considerations in the design of this study, including early initiation of rescue therapy, limit the extent to which our findings can be extrapolated to endemic settings. For example, a proportion of individuals with subclinical bacteremia could have progressed to symptomatic disease had antibiotic treatment been delayed, as was observed in historical typhoid challenge studies (W. E. Woodward, unpublished monograph). As only a single strain was used in this study, we cannot conclusively rule out a strain-specific effect for our observations. However, the NVGH308 strain is a recent clinical isolate from a symptomatic case with bacteremia, and is closely related to currently circulating strains in South Asia. Additionally, *S*. Paratyphi A is a clonal monomorphic pathogen containing limited genomic variation, suggesting that the pathogenicity and immune response to the NVGH308 strain will translate to other wild-type strains [27]. Future work could identify whether these observations apply to other strains of *S.* Paratyphi A and also investigate B and C strains in the human challenge model. The evaluation of only 2 challenge doses is one potential limitation of this study, and challenge at of higher dose ranges and/or rechallenge studies could be undertaken to better define clinical outcomes.

In summary, we have established the first *S*. Paratyphi A human challenge model and have described the clinical and microbiological response to challenge in healthy adult volunteers, presenting marked differences from those previously seen with *S*. Typhi. While *S*. Typhi challenge models have existed for several decades, the need for a *S*. Paratyphi A challenge model arguably exceeds that for *S*. Typhi, as far less is understood regarding the responses to infection and there are no specific control measures yet available. Development of a successful paratyphoid challenge model could offer distinctive insights into paratyphoid disease as well as providing a platform to expedite the development and implementation of much-needed vaccines and diagnostics.

## Supplementary Material

Supplementary Data

## Notes

***Acknowledgments.*** The study team wishes to acknowledge the contribution of study participants and the assistance of the following: Public Health England, Oxfordshire; Oxford University Hospital Laboratories; clinical and laboratory staff at Oxford Vaccine Group; University of Maryland for provision of antigens; and Emergent BioSolutions for provision of standard sera to complete the antibody work.

***Disclaimer.*** The study funders are independent of the study design, study management, analyses, and interpretation of the study results.

***Financial support.*** This work was supported by the the Bill & Melinda Gates Foundation (reference OPP1084259) and the European Vaccine Initiative (reference PIM). The typhoid challenge study was supported by a Wellcome Trust Strategic Translational Award (grant number 092661). The Oxford Vaccine Group, University of Oxford, is supported by the National Institute for Health Research Clinical Research Network. L. B. M. reports grants from the Wellcome Trust outside of the submitted work. A. J. P., T. C. D., and C. J. B. are supported by the National Institute for Health Research Oxford Biomedical Research Centre (Translational Research Fellowship to T. C. D.). C. J. B. is supported by the European Union (FP7) as a Marie Curie fellow.

***Potential conflicts of interest.*** L. B. M. and A. P. are employees of the GSK group of companies, which has partnered with Biological E (Hyderabad, India) for development of a bivalent typhoid and paratyphoid A vaccine. GSK Vaccines for Global Health has received a grant from the Wellcome Trust for development of a bivalent typhoid/paratyphoid A vaccine. M. M. L. reports a license agreement between the University of Maryland and Bharat Biotech for a bivalent typhoid/paratyphoid vaccine in early preclinical development, for which he is a coinventor. A. J. P. has previously undertaken clinical studies on behalf of the University of Oxford, which were funded by vaccine manufacturers, but no longer does so. His department has received unrestricted educational grants from vaccine manufacturers to support delivery of a course on childhood infection. All other authors report no potential conflicts. All authors have submitted the ICMJE Form for Disclosure of Potential Conflicts of Interest. Conflicts that the editors consider relevant to the content of the manuscript have been disclosed.
