# Supplementary material for: Evaluation of the Clinical and Microbiological Response to Salmonella Paratyphi A Infection in the First Paratyphoid Human Challenge Model
Source: Clin Infect Dis. 2017 Feb 4;64(8):1066–73. doi: 10.1093/cid/cix042 (PMC5439345; doi:10.1093/cid/cix042)
Supplement: Supplementary Data [file cix042_Supplementary_Data.zip › Supplementary_Material_Dobinson_et_al_CID_V2.docx]

**Supplementary Methods**

**Safety monitoring**

An independent Data Safety Monitoring Committee (DSMC) was established, who had access to all data. Approvals were obtained from the DSMC prior to any protocol amendments and dose alterations. The study (ClinicalTrials.gov identifier NCT02100397) was approved by Oxfordshire Research Ethics Committee A (14/SC/0004) and performed according to the provisions of the Declaration of Helsinki and Good Clinical Practice guidelines.

**Challenge strain genome sequencing**

Whole genome sequencing was undertaken to identify the phylogenetic relationship of NVGH308 to other circulating strains (**Supplementary Figure 1**). Genomic DNA from representative *S*. Paratyphi A isolates were extracted using the Wizard genome DNA extraction kit (Promega, USA) and sequenced using the Illumina Hiseq platform to generate 75 paired-end reads. Reads were mapped to the reference sequence of S. Paratyphi A strain AKU_12601 (accession no: FM200053) using SMALT (version 0.7.4). Single nucleotide polymorphisms (SNPs) in the core genomes were called and filtered using SAMtools.[2,3] A maximum likelihood (ML) phylogeny was estimated from the SNP alignment (1,539 SNPs sites) using RAxML (version 7.8.6) with the generalized time-reversible substitution model (GTR) and a gamma distribution, with support for the phylogeny assessed via 100 bootstrap replicates.

**Challenge procedure**

The challenge inoculum was freshly prepared on each day of challenge by diluting frozen stock vials of GMP NVGH308 strain. The challenge inoculum was suspended in a NaHCO_3_ solution (0.53g/30ml) and was kept on ice prior to administration within two hours of preparation. Participants fasted for 90 minutes prior to challenge and drank a NaHCO_3_ solution (2.1g/120ml) two minutes prior to ingestion of challenge inoculum.

The dose administered (‘actual dose’) was calculated by direct plating of aliquots on Tryptone soya agar (TSA) plates (Oxoid/PO0163A) in triplicate and colony counting after 24 hours incubation at 37°C with 5%CO_2_.

**Microbiology**

Blood cultures (10ml volume, BACTEC Plus Aerobic vials, BD) and stool cultures were collected daily.[11] Quantitative blood culture samples were collected immediately prior to antibiotic treatment by inoculation of 10 mL blood into an ISOLATOR 10 tube (Alere, UK).[11]

**ELISA**

O-antigen was extracted by GVGH from *S*. Paratyphi NVGH308 GMP Master Cell Bank fermented at 30 L as previously described.[4] The O-antigen was fully characterized for O-antigen content, O-antigen size, sugar composition, O-acetylation level and impurities. H-antigen was purified from *S*. Paratyphi CVD 1902 (University of Maryland, USA).[5] Nominal ELISA units were calculated based on absorbance values relative to standard curves derived from pooled serum (Emergent BioSolutions, USA; from the strongest responders to O-antigen following vaccination with M01ZH09), which had been standardised across all ELISA to reach the same range of absorbance values within identical conditions and in which 1 ELISA unit was set at the highest standard concentration.

**Statistical Analysis**

Confidence intervals for the proportion of participants who met diagnostic end-points were calculated using the Clopper-Pearson exact method. Serology data were log_2_-transformed prior to analysis and analysed using linear regression models adjusting for baseline values and challenge dose. The anti-log of the parameter estimates are presented as adjusted geometric mean ratios with associated 95% CI. All participants recorded symptom scores for 21 days post challenge and these were summed across all days to obtain a total score for analysis.

Total symptom scores were compared between participants with *S.* Paratyphi A and those challenged with *S.* Typhi participants using a Mann Whitney U test. Fisher’s exact test was used to compare binary endpoints. The number of days with positive stool cultures and positive blood cultures were compared between *S*. Paratyphi A and *S*. Typhi challenged participants in Poisson regression models with robust standard errors. The log of the number of days with samples provided by each participant was included as an offset in the model. Results are presented as incidence rate ratios (IRR) (*S.* Paratyphi A/*S.* Typhi) and associated 95% confidence interval.

***Supplementary Table 1***

Detailed antibiogram of the *S.* Paratyphi A NVGH308 strain.

| **Sensitivity Results^a^** | | |
| --- | --- | --- |
|  | **Disc diffusion** | **MIC^b^ (μg/ml)** |
| Ampicillin | Sensitive | 2.0=Sensitive |
| Cefalexin | Sensitive |  |
| Augmentin | Sensitive |  |
| Ceftriaxone | Sensitive |  |
| Gentamicin | Sensitive |  |
| Amikacin | Sensitive |  |
| Tobramycin | Sensitive |  |
| Ceftazidime | Sensitive |  |
| Meropenem | Sensitive |  |
| Ertapenem | Sensitive |  |
| Ciprofloxacin |  | 0.06=Sensitive |

1. Disk diffusion assays and broth dilution assays, as per CLSI guidelines.[6]
2. MIC = Minimal inhibitory concentration in broth dilution experiments.

***Supplementary Table 2***

Detailed clinical symptom profiles following *S*. Paratyphi A challenge. Solicited systemic symptoms were recorded by participants for 21 days using an online diary.

| **Symptom** | **Dose** | **Outcome ^a^** | **Number (%) affected** | **Maximum severity ^b^** | | | | **Number of symptomatic days, Median**  **(Range)** | **Symptom score, Median (Range)** | **OR**  **(95% CI)^c^** |
| --- | --- | --- | --- | --- | --- | --- | --- | --- | --- | --- |
|  |  |  |  | **None** | **Mild** | **Moderate** | **Severe** |  |  |  |
| **Headache** |  |  |  |  |  |  |  |  |  | 5.67(1.25 to 21.77) |
|  | Group 1 | Paratyphoid diagnosed | 11(92) | 8.3% | 25.0% | 50.0% | 16.7% | 5(0-8) | 6.5(0-15) |  |
|  | (1-5x10^3^ CFU) | Paratyphoid NOT diagnosed | 3(38) | 62.5% | 25.0% | 0.0% | 12.5% | 0(0-5) | 0(0-12) |  |
|  |  |  |  |  |  |  |  |  |  |  |
|  |  |  |  |  |  |  |  |  |  |  |
|  | Group 2 | Paratyphoid diagnosed | 6(75) | 25.0% | 25.0% | 25.0% | 25.0% | 2.5(0-11) | 2.5(0-22) |  |
|  | (0.5-1x 10^3^CFU) | Paratyphoid NOT diagnosed | 7(58) | 41.7% | 50.0% | 0.0% | 8.3% | 1(0-9) | 1(0-20) |  |
|  |  |  |  |  |  |  |  |  |  |  |
|  |  |  |  |  |  |  |  |  |  |  |
|  |  | All Paratyphoid diagnosed | 17(85) | 15.0% | 25.0% | 40.0% | 20.0% | 3.5(0-11) | 5(0-22) |  |
| **Malaise** |  |  |  |  |  |  |  |  |  | 7(1.81 to 31.3) |
|  | Group 1 | Paratyphoid diagnosed | 11(92) | 8.3% | 25.0% | 33.3% | 33.3% | 5.5(0-11) | 9(0-18) |  |
|  | (1-5x10^3^ CFU) | Paratyphoid NOT diagnosed | 1(12.5) | 87.5% | 12.5% | 0.0% | 0.0% | 0(0-4) | 0(0-4) |  |
|  |  |  |  |  |  |  |  |  |  |  |
|  |  |  |  |  |  |  |  |  |  |  |
|  | Group 2 | Paratyphoid diagnosed | 4(50) | 50.0% | 0.0% | 50.0% | 0.0% | 0.5(0-6) | 1(0-8) |  |
|  | (0.5-1x 10^3^CFU) | Paratyphoid NOT diagnosed | 5(42) | 58.3% | 16.7% | 16.7% | 8.3% | 0(0-9) | 0(0-21) |  |
|  |  |  |  |  |  |  |  |  |  |  |
|  |  |  |  |  |  |  |  |  |  |  |
|  |  | All Paratyphoid diagnosed | 15(75) | 25.0% | 15.0% | 40.0% | 20.0% | 3(0-11) | 4(0-18) |  |
| **Abdominal pain** |  |  |  |  |  |  |  |  |  | 7(1.81 to 31.3) |
|  | Group 1 | Paratyphoid diagnosed | 9(75) | 25.0% | 33.3% | 33.3% | 8.3% | 3(0-10) | 3(0-15) |  |
|  | (1-5x10^3^ CFU) | Paratyphoid NOT diagnosed | 2(25) | 75.0% | 25.0% | 0.0% | 0.0% | 0(0-3) | 0(0-3) |  |
|  |  |  |  |  |  |  |  |  |  |  |
|  |  |  |  |  |  |  |  |  |  |  |
|  | Group 2 | Paratyphoid diagnosed | 5(63) | 37.5% | 50.0% | 12.5% | 0.0% | 1(0-5) | 1(0-5) |  |
|  | (0.5-1x 10^3^CFU) | Paratyphoid NOT diagnosed | 3(25) | 75.0% | 8.3% | 16.7% | 0.0% | 0(0-7) | 0(0-8) |  |
|  |  |  |  |  |  |  |  |  |  |  |
|  |  |  |  |  |  |  |  |  |  |  |
|  |  | All Paratyphoid diagnosed | 14(70) | 30.0% | 40.0% | 25.0% | 5.0% | 1.5(0-10) | 1.5(0-15) |  |
| **Myalgia** |  |  |  |  |  |  |  |  |  | 4.33(1.22 to 15.12) |
|  | Group 1 | Paratyphoid diagnosed | 10(83) | 16.7% | 58.3% | 16.7% | 8.3% | 2(0-8) | 2(0-10) |  |
|  | (1-5x10^3^ CFU) | Paratyphoid NOT diagnosed | 2(25) | 75.0% | 12.5% | 0.0% | 12.5% | 0(0-4) | 0(0-4) |  |
|  |  |  |  |  |  |  |  |  |  |  |
|  |  |  |  |  |  |  |  |  |  |  |
|  | Group 2 | Paratyphoid diagnosed | 4(50) | 50.0% | 25.0% | 25.0% | 0.0% | 0.5(0-4) | 0.5(0-5) |  |
|  | (0.5-1x 10^3^CFU) | Paratyphoid NOT diagnosed | 5(42) | 58.3% | 33.3% | 8.3% | 0.0% | 0(0-6) | 0(0-7) |  |
|  |  |  |  |  |  |  |  |  |  |  |
|  |  |  |  |  |  |  |  |  |  |  |
|  |  | All Paratyphoid diagnosed | 14(70) | 30.0% | 45.0% | 20.0% | 5.0% | 2(0-8) | 2(0-10) |  |
| **Anorexia** |  |  |  |  |  |  |  |  |  | 8.5(1.89 to 32.45) |
|  | Group 1 | Paratyphoid diagnosed | 8(67) | 33.3% | 25.0% | 33.3% | 8.3% | 2.5(0-7) | 3(0-8) |  |
|  | (1-5x10^3^ CFU) | Paratyphoid NOT diagnosed | 1(13) | 87.5% | 12.5% | 0.0% | 0.0% | 0(0-1) | 0(0-1) |  |
|  |  |  |  |  |  |  |  |  |  |  |
|  |  |  |  |  |  |  |  |  |  |  |
|  | Group 2 | Paratyphoid diagnosed | 4(50) | 50.0% | 25.0% | 12.5% | 12.5% | 1(0-9) | 1(0-15) |  |
|  | (0.5-1x 10^3^CFU) | Paratyphoid NOT diagnosed | 2(17) | 83.3% | 8.3% | 8.3% | 0.0% | 0(0-6) | 0(0-10) |  |
|  |  |  |  |  |  |  |  |  |  |  |
|  |  |  |  |  |  |  |  |  |  |  |
|  |  | All Paratyphoid diagnosed | 12(60) | 40.0% | 25.0% | 25.0% | 10.0% | 2(0-9) | 2(0-15) |  |
| **Arthralgia** |  |  |  |  |  |  |  |  |  | 13.5(2.43 to 66.45) |
|  | Group 1 | Paratyphoid diagnosed | 9(75) | 25.0% | 41.7% | 25.0% | 8.3% | 1.5(0-7) | 2(0-9) |  |
|  | (1-5x10^3^ CFU) | Paratyphoid NOT diagnosed | 1(13) | 87.5% | 12.5% | 0.0% | 0.0% | 0(0-2) | 0(0-2) |  |
|  |  |  |  |  |  |  |  |  |  |  |
|  |  |  |  |  |  |  |  |  |  |  |
|  | Group 2 | Paratyphoid diagnosed | 3(38) | 62.5% | 12.5% | 25.0% | 0.0% | 0(0-5) | 0(0-6) |  |
|  | (0.5-1x 10^3^CFU) | Paratyphoid NOT diagnosed | 1(8) | 91.7% | 8.3% | 0.0% | 0.0% | 0(0-2) | 0(0-2) |  |
|  |  |  |  |  |  |  |  |  |  |  |
|  |  |  |  |  |  |  |  |  |  |  |
|  |  | All Paratyphoid diagnosed | 12(60) | 40.0% | 30.0% | 25.0% | 5.0% | 1(0-7) | 1(0-9) |  |
| **Constipation** |  |  |  |  |  |  |  |  |  | 0.82(0.24 to 2.72) |
|  | Group 1 | Paratyphoid diagnosed | 5(42) | 58.3% | 8.3% | 33.3% | 0.0% | 0(0-9) | 0(0-14) |  |
|  | (1-5x10^3^ CFU) | Paratyphoid NOT diagnosed | 3(38) | 62.5% | 25.0% | 12.5% | 0.0% | 0(0-2) | 0(0-3) |  |
|  |  |  |  |  |  |  |  |  |  |  |
|  |  |  |  |  |  |  |  |  |  |  |
|  | Group 2 | Paratyphoid diagnosed | 5(63) | 37.5% | 50.0% | 12.5% | 0.0% | 2(0-4) | 2(0-4) |  |
|  | (0.5-1x 10^3^CFU) | Paratyphoid NOT diagnosed | 8(67) | 33.3% | 66.7% | 0.0% | 0.0% | 1(0-3) | 1(0-3) |  |
|  |  |  |  |  |  |  |  |  |  |  |
|  |  |  |  |  |  |  |  |  |  |  |
|  |  | All Paratyphoid diagnosed | 10(50) | 50.0% | 25.0% | 25.0% | 0.0% | 0.5(0-9) | 0.5(0-14) |  |
| **Nausea/vomiting** |  |  |  |  |  |  |  |  |  | 3.9(0.73 to 20.37) |
|  | Group 1 | Paratyphoid diagnosed | 3(25) | 75.0% | 16.7% | 8.3% | 0.0% | 0(0-5) | 0(0-5) |  |
|  | (1-5x10^3^ CFU) | Paratyphoid NOT diagnosed | 1(13) | 87.5% | 12.5% | 0.0% | 0.0% | 0(0-1) | 0(0-1) |  |
|  |  |  |  |  |  |  |  |  |  |  |
|  |  |  |  |  |  |  |  |  |  |  |
|  | Group 2 | Paratyphoid diagnosed | 3(38) | 62.5% | 37.5% | 0.0% | 0.0% | 0(0-4) | 0(0-4) |  |
|  | (0.5-1x 10^3^CFU) | Paratyphoid NOT diagnosed | 1(8) | 91.7% | 8.3% | 0.0% | 0.0% | 0(0-7) | 0(0-7) |  |
|  |  |  |  |  |  |  |  |  |  |  |
|  |  |  |  |  |  |  |  |  |  |  |
|  |  | All Paratyphoid diagnosed | 6(30) | 70.0% | 25.0% | 5.0% | 0.0% | 0(0-5) | 0(0-5) |  |
| **Cough** |  |  |  |  |  |  |  |  |  | 2.3(0.45 to 12.8) |
|  | Group 1 | Paratyphoid diagnosed | 3(25) | 75.0% | 25.0% | 0.0% | 0.0% | 0(0-9) | 0(0-9) |  |
|  | (1-5x10^3^ CFU) | Paratyphoid NOT diagnosed | 0(0) | 100.0% | 0.0% | 0.0% | 0.0% | 0(0-0) | 0(0-0) |  |
|  |  |  |  |  |  |  |  |  |  |  |
|  |  |  |  |  |  |  |  |  |  |  |
|  | Group 2 | Paratyphoid diagnosed | 1(13) | 87.5% | 12.5% | 0.0% | 0.0% | 0(0-3) | 0(0-3) |  |
|  | (0.5-1x 10^3^CFU) | Paratyphoid NOT diagnosed | 2(17) | 83.3% | 16.7% | 0.0% | 0.0% | 0(0-3) | 0(0-3) |  |
|  |  |  |  |  |  |  |  |  |  |  |
|  |  |  |  |  |  |  |  |  |  |  |
|  |  | All Paratyphoid diagnosed | 4(20) | 80.0% | 20.0% | 0.0% | 0.0% | 0(0-9) | 0(0-9) |  |
| **Diarrhoea** |  |  |  |  |  |  |  |  |  | 0.6(0.10 to 3.43) |
|  | Group 1 | Paratyphoid diagnosed | 0(0) | 100.0% | 0.0% | 0.0% | 0.0% | 0(0-0) | 0(0-0) |  |
|  | (1-5x103 CFU) | Paratyphoid NOT diagnosed | 0(0) | 100.0% | 0.0% | 0.0% | 0.0% | 0(0-0) | 0(0-0) |  |
|  |  |  |  |  |  |  |  |  |  |  |
|  |  |  |  |  |  |  |  |  |  |  |
|  | Group 2 | Paratyphoid diagnosed | 2(25) | 75.0% | 0.0% | 0.0% | 25.0% | 0(0-1) | 0(0-3) |  |
|  | (0.5-1x 103CFU) | Paratyphoid NOT diagnosed | 3(25) | 75.0% | 0.0% | 8.3% | 16.7% | 0(0-4) | 0(0-12) |  |
|  |  |  |  |  |  |  |  |  |  |  |
|  |  |  |  |  |  |  |  |  |  |  |
|  |  | All Paratyphoid diagnosed | 2(10) | 90.0% | 0.0% | 0.0% | 10.0% | 0(0-1) | 0(0-3) |  |
| **Rash** |  |  |  |  |  |  |  |  |  | 1 |
|  | Group 1 | Paratyphoid diagnosed | 0 (0) | 100.0% | 0.0% | 0.0% | 0.0% | 0(0-0) | 0(0-0) |  |
|  | (1-5x103 CFU) | Paratyphoid NOT diagnosed | 0 (0) | 100.0% | 0.0% | 0.0% | 0.0% | 0(0-0) | 0(0-0) |  |
|  |  |  |  |  |  |  |  |  |  |  |
|  |  |  |  |  |  |  |  |  |  |  |
|  | Group 2 | Paratyphoid diagnosed | 0 (0) | 100.0% | 0.0% | 0.0% | 0.0% | 0(0-0) | 0(0-0) |  |
|  | (0.5-1x 103CFU) | Paratyphoid NOT diagnosed | 0 (0) | 100.0% | 0.0% | 0.0% | 0.0% | 0(0-0) |  |  |
|  |  |  |  |  |  |  |  |  |  |  |
|  |  |  |  |  |  |  |  |  |  |  |
|  |  | All Paratyphoid diagnosed | 0 (0) | 100.0% | 0.0% | 0.0% | 0.0% | 0(0-0) | 0(0-0) |  |
|  |  |  |  |  |  |  |  |  |  |  |

1. Paratyphoid diagnosed = *S*. Paratyphi A bacteraemia or temperature ≥38°C for ≥12 hours. Paratyphoid NOT diagnosed = Pre-specified diagnostic criteria not met.
2. Mild = Present but no limitation of usual activity; Moderate =some limitation of daily activity; Severe = unable to perform normal daily activity.
3. Odds ratios compare presence or absence of symptoms in participants diagnosed with paratyphoid compared with participants not diagnosed.

***Supplementary Table 3***

Clinical symptom profiles (Day 0 to Day 14) following *S*. Typhi and *S*. Paratyphi A challenge. Solicited systemic symptoms were recorded by participants for up to 21 days using a diary card.

| **Challenge Agent ^b^** | | | |
| --- | --- | --- | --- |
| **Symptom ^a^** | ***S.* Typhi** | ***S*. Paratyphi A** | **Odds Ratio (95% CI)^c^** |
| **Headache** |  |  |  |
|  | 1.71(0.61 to 4.80) | | |
| Any symptomatic days, n (%) |  |  |  |
| Enteric fever diagnosed | 23(96) | 17(85) |  |
| Enteric fever NOT diagnosed | 10(63) | 10(50) |  |
| All | 33(82.5) | 27(67.5) |  |
|  |  |  |  |
| Any severe (Grade 3 ) symptoms |  |  |  |
| Enteric fever diagnosed | 7(29) | 4(20) |  |
| Enteric fever NOT diagnosed | 1(6.25) | 2(10) |  |
|  |  |  |  |
| No. of symptomatic days, median(range) |  |  |  |
| Enteric fever diagnosed | 4.5(0-10) | 3.5(0-11) |  |
| Enteric fever NOT diagnosed | 1(0-4) | 0(0-1) |  |
|  |  |  |  |
| Symptom score, median(range) |  |  |  |
| Enteric fever diagnosed | 7(0-18) | 5(0-22) |  |
| Enteric fever NOT diagnosed | 1(0-7) | 0.5(0-20) |  |
| **Malaise** |  |  |  |
|  | 1.73(0.69 to 4.34) | | |
| Any symptomatic days, n (%) |  |  |  |
| Enteric fever diagnosed | 23(96) | 15(75) |  |
| Enteric fever NOT diagnosed | 7(44) | 6(30) |  |
| All | 30(75) | 21(52.5) |  |
|  |  |  |  |
| Any severe (Grade 3 ) symptoms |  |  |  |
| Enteric fever diagnosed | 8(33) | 4(20) |  |
| Enteric fever NOT diagnosed | 3(18.75) | 1(5) |  |
|  |  |  |  |
| No. of symptomatic days, median(range) |  |  |  |
| Enteric fever diagnosed | 5(0-15) | 3(0-11) |  |
| Enteric fever NOT diagnosed | 0(0-4) | 0(0-9) |  |
|  |  |  |  |
| Symptom score, median(range) |  |  |  |
| Enteric fever diagnosed | 8.5(0-18) | 4(0-18) |  |
| Enteric fever NOT diagnosed | 0(0-6) | 0(0-21) |  |
| **Abdominal pain** |  |  |  |
|  | 1.84(0.76 to 4.49) | | |
| Any symptomatic days, n (%) |  |  |  |
| Enteric fever diagnosed | 20(83) | 14(70) |  |
| Enteric fever NOT diagnosed | 6(38) | 5(25) |  |
| All | 26(65) | 19(47.5) |  |
|  |  |  |  |
| Any severe (Grade 3 ) symptoms |  |  |  |
| Enteric fever diagnosed | 2(8) | 1(5) |  |
| Enteric fever NOT diagnosed | 1(6.25) | 0(0) |  |
|  |  |  |  |
| No. of symptomatic days, median(range) |  |  |  |
| Enteric fever diagnosed | 3(0-12) | 1.5(0-10) |  |
| Enteric fever NOT diagnosed | 0(0-2) | 0(0-7) |  |
| Symptom score, median(range) |  |  |  |
| Enteric fever diagnosed | 4(0-13) | 1.5(0-15) |  |
| Enteric fever NOT diagnosed | 0(0-3) | 0(0-8) |  |
| **Myalgia** |  |  |  |
|  | 1.68(0.68 to 4.13) | | |
| Any symptomatic days, n (%) |  |  |  |
| Enteric fever diagnosed | 21(88) | 14(70) |  |
| Enteric fever NOT diagnosed | 7(44) | 7(35) |  |
| All | 28(70) | 21(52.5) |  |
|  |  |  |  |
| Any severe (Grade 3 ) symptoms |  |  |  |
| Enteric fever diagnosed | 5(21) | 1(5) |  |
| Enteric fever NOT diagnosed | 2(12.5) | 1(5) |  |
|  |  |  |  |
| No. of symptomatic days, median(range) |  |  |  |
| Enteric fever diagnosed | 3.5(0-9) | 2(0-8) |  |
| Enteric fever NOT diagnosed | 0(0-4) | 0(0-6) |  |
|  |  |  |  |
| Symptom score, median(range) |  |  |  |
| Enteric fever diagnosed | 5(0-18) | 2(0-10) |  |
| Enteric fever NOT diagnosed | 0(0-8) | 0(0-7) |  |
| **Anorexia** |  |  |  |
|  | 1.39(0.55 to 3.46) | | |
| Any symptomatic days, n (%) |  |  |  |
| Enteric fever diagnosed | 22(92) | 12(60) |  |
| Enteric fever NOT diagnosed | 5(31) | 3(15) |  |
| All | 27(67.5) | 15(37.5) |  |
|  |  |  |  |
| Any severe (Grade 3 ) symptoms |  |  |  |
| Enteric fever diagnosed | 6(25) | 2(10) |  |
| Enteric fever NOT diagnosed | 2(12.5) | 0(0) |  |
|  |  |  |  |
| No. of symptomatic days, median(range) |  |  |  |
| Enteric fever diagnosed | 4(0-10) | 2(0-9) |  |
| Enteric fever NOT diagnosed | 0(0-4) | 0(0-6) |  |
|  |  |  |  |
| Symptom score, median(range) |  |  |  |
| Enteric fever diagnosed | 6(0-14) | 2(0-15) |  |
| Enteric fever NOT diagnosed | 0(0-4) | 0(0-10) |  |
| **Arthralgia** |  |  |  |
|  | 1.68(0.68 to 4.13) | | |
| Any symptomatic days, n (%) |  |  |  |
| Enteric fever diagnosed | 19(79) | 12(60) |  |
| Enteric fever NOT diagnosed | 2(13) | 2(10) |  |
| All | 21(52.5) | 14(35) |  |
|  |  |  |  |
| Any severe (Grade 3 ) symptoms |  |  |  |
| Enteric fever diagnosed | 4(17) | 1(5) |  |
| Enteric fever NOT diagnosed | 1(6.25) | 0(0) |  |
|  |  |  |  |
| No. of symptomatic days, median(range) |  |  |  |
| Enteric fever diagnosed | 3(0-8) | 1(0-7) |  |
| Enteric fever NOT diagnosed | 0(0-4) | 0(0-2) |  |
|  |  |  |  |
| Symptom score, median(range) |  |  |  |
| Enteric fever diagnosed | 5(0-18) | 1(0-9) |  |
| Enteric fever NOT diagnosed | 0(0-6) | 0(0-2) |  |
| **Constipation** |  |  |  |
|  | 1.11(0.46 to 2.67) | | |
| Any symptomatic days, n (%) |  |  |  |
| Enteric fever diagnosed | 16(67) | 10(50) |  |
| Enteric fever NOT diagnosed | 6(38) | 11(55) |  |
| All | 22(55) | 21(52.5) |  |
|  |  |  |  |
| Any severe (Grade 3 ) symptoms |  |  |  |
| Enteric fever diagnosed | 2(8) | 0(0) |  |
| Enteric fever NOT diagnosed | 0(0) | 0(0) |  |
|  |  |  |  |
| No. of symptomatic days, median(range) |  |  |  |
| Enteric fever diagnosed | 1(0-9) | 0.5(0-9) |  |
| Enteric fever NOT diagnosed | 0(0-8) | 1(0-3) |  |
|  |  |  |  |
| Symptom score, median(range) |  |  |  |
| Enteric fever diagnosed | 1.5(0-12) | 0.5(0-14) |  |
| Enteric fever NOT diagnosed | 0(0-9) | 1(0-3) |  |
| **Nausea/vomiting** |  |  |  |
|  | 5.74(2.15 to 15.30) | | |
| Any symptomatic days, n (%) |  |  |  |
| Enteric fever diagnosed | 20(83) | 6(30) |  |
| Enteric fever NOT diagnosed | 5(31) | 2(10) |  |
| All | 25(62.5) | 8(20) |  |
|  |  |  |  |
| Any severe (Grade 3 ) symptoms |  |  |  |
| Enteric fever diagnosed | 10(42) | 0(0) |  |
| Enteric fever NOT diagnosed | 2(12.5) | 0(0) |  |
|  |  |  |  |
| No. of symptomatic days, median(range) |  |  |  |
| Enteric fever diagnosed | 2.5(0-12) | 0(0-5) |  |
| Enteric fever NOT diagnosed | 0(0-1) | 0(0-7) |  |
|  |  |  |  |
| Symptom score, median(range) |  |  |  |
| Enteric fever diagnosed | 0(0-2) | 0(0-5) |  |
| Enteric fever NOT diagnosed | 0(0-3) | 0(0-7) |  |
|  |  |  |  |
| **Cough** |  |  |  |
|  | 4.27(1.53 to 11.89) | | |
| Any symptomatic days, n (%) |  |  |  |
| Enteric fever diagnosed | 16(67) | 4(20) |  |
| Enteric fever NOT diagnosed | 4(25) | 2(10) |  |
| All | 20(50) | 6(15) |  |
|  |  |  |  |
| Any severe (Grade 3 ) symptoms |  |  |  |
| Enteric fever diagnosed | 0(0) | 0(0) |  |
| Enteric fever NOT diagnosed | 0(0) | 0(0) |  |
|  |  |  |  |
| No. of symptomatic days, median(range) |  |  |  |
| Enteric fever diagnosed | 2(0-9) | 0(0-9) |  |
| Enteric fever NOT diagnosed | 0(0-10) | 0(0-3) |  |
|  |  |  |  |
| Symptom score, median(range) |  |  |  |
| Enteric fever diagnosed | 2(0-9) | 0(0-9) |  |
| Enteric fever NOT diagnosed | 0(0-10) | 0(0-3) |  |
| **Diarrhoea** |  |  |  |
|  | 2.27(0.79 to 6.49) | | |
| Any symptomatic days, n (%) |  |  |  |
| Enteric fever diagnosed | 9(38) | 2(10) |  |
| Enteric fever NOT diagnosed | 4(25) | 3(15) |  |
| All | 13(32.5) | 5(12.5) |  |
|  |  |  |  |
| Any severe (Grade 3 ) symptoms |  |  |  |
| Enteric fever diagnosed | 9(38) | 2(10) |  |
| Enteric fever NOT diagnosed | 0(0) | 2(10) |  |
|  |  |  |  |
| No. of symptomatic days, median(range) |  |  |  |
| Enteric fever diagnosed | 0(0-4) | 0(0-1) |  |
| Enteric fever NOT diagnosed | 0(0-1) | 0(0-4) |  |
|  |  |  |  |
| Symptom score, median(range) |  |  |  |
| Enteric fever diagnosed | 0(0-7) | 0(0-3) |  |
| Enteric fever NOT diagnosed | 0(0-2) | 0(0-12) |  |
|  |  |  |  |

1. Enteric fever diagnosed = *S*. Typhi/Paratyphi A bacteraemia or temperature ≥38°C for ≥12 hours. Enteric fever NOT diagnosed = Pre-specified diagnostic criteria not met. Mild = Present but no limitation of usual activity; Moderate =some limitation of daily activity; Severe = unable to perform normal daily activity.
2. Challenge was performed at a dose of either 1-5 x 10^3^ CFU (n=20) or 1-5 x 10^4^ CFU (n=20) *S*. Typhi and 1-5 x 10^3^ CFU (n=20) or 0.5 -1 x 10^3^ CFU (n=20) for *S*. Paratyphi A.
3. Odds ratios compare presence or absence of symptoms in participants diagnosed with *S*. Typhi infection compared with *S*. Paratyphi infection.

**Supplementary Figure 1**

The phylogenetic positioning of the *Salmonella* *enterica* serovar Paratyphi A challenge strain NVGH308. Maximum Likelihood phylogeny generated from 1,539 SNPs (Single Nucleotide Polymorphisms) identified in the whole genome sequences of 42 previously sequenced global isolated of S. Paratyphi A isolates [28]. The NVGH308 challenge strain (labelled ED199) is highlighted in red; strain names are expanded to show the location and time of isolation. The scale bar indicates the number of substitution per variable site.


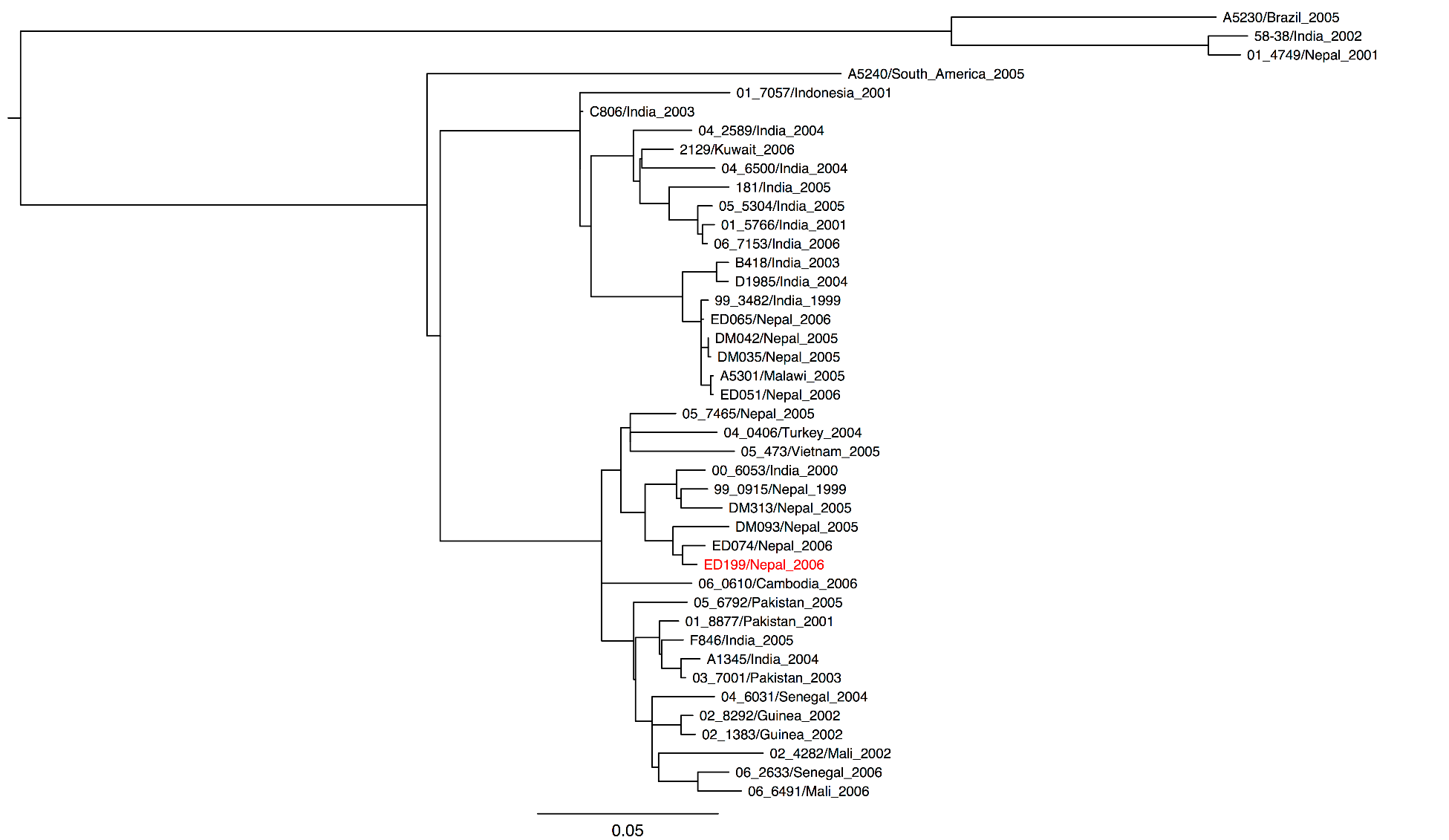


***Supplementary Figure 2*** Participant recruitment and enrolment flow diagram.


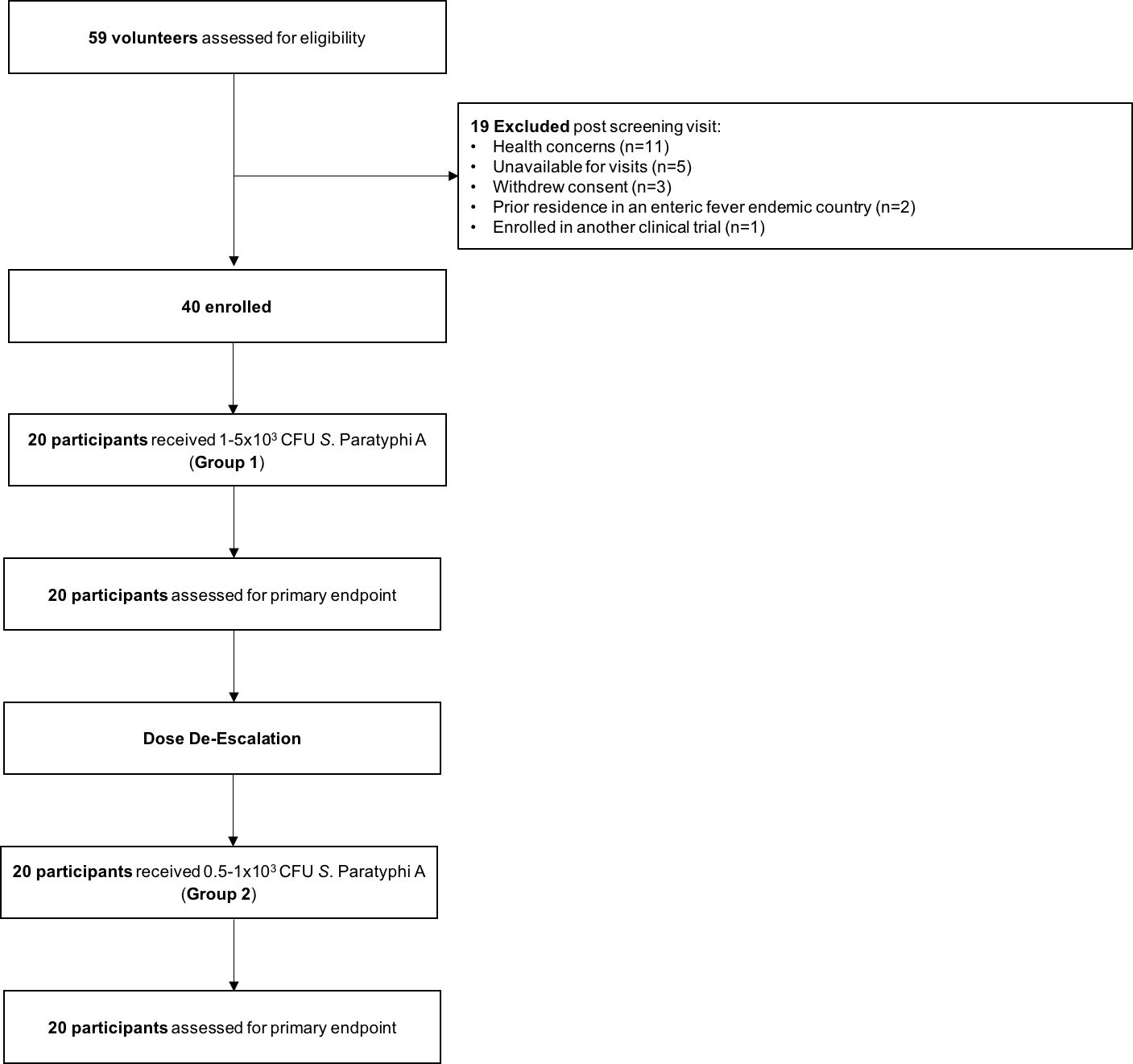


***Supplementary Figure 3*** Laboratory parameters (median, range) for participants with acute paratyphoid disease from Group 1 and Group 2 (n=20). **(a)** Haematological parameters; **(b)** Biochemistry. Grey lines indicate reference ranges. Peripheral blood haematological and biochemical analysis were performed on alternate days after challenge and up to 96 hours after paratyphoid diagnosis, and included full blood count, renal profile, liver function tests and C-reactive protein. PD= Day of Paratyphoid diagnosis, defined as first day of bacteraemia.


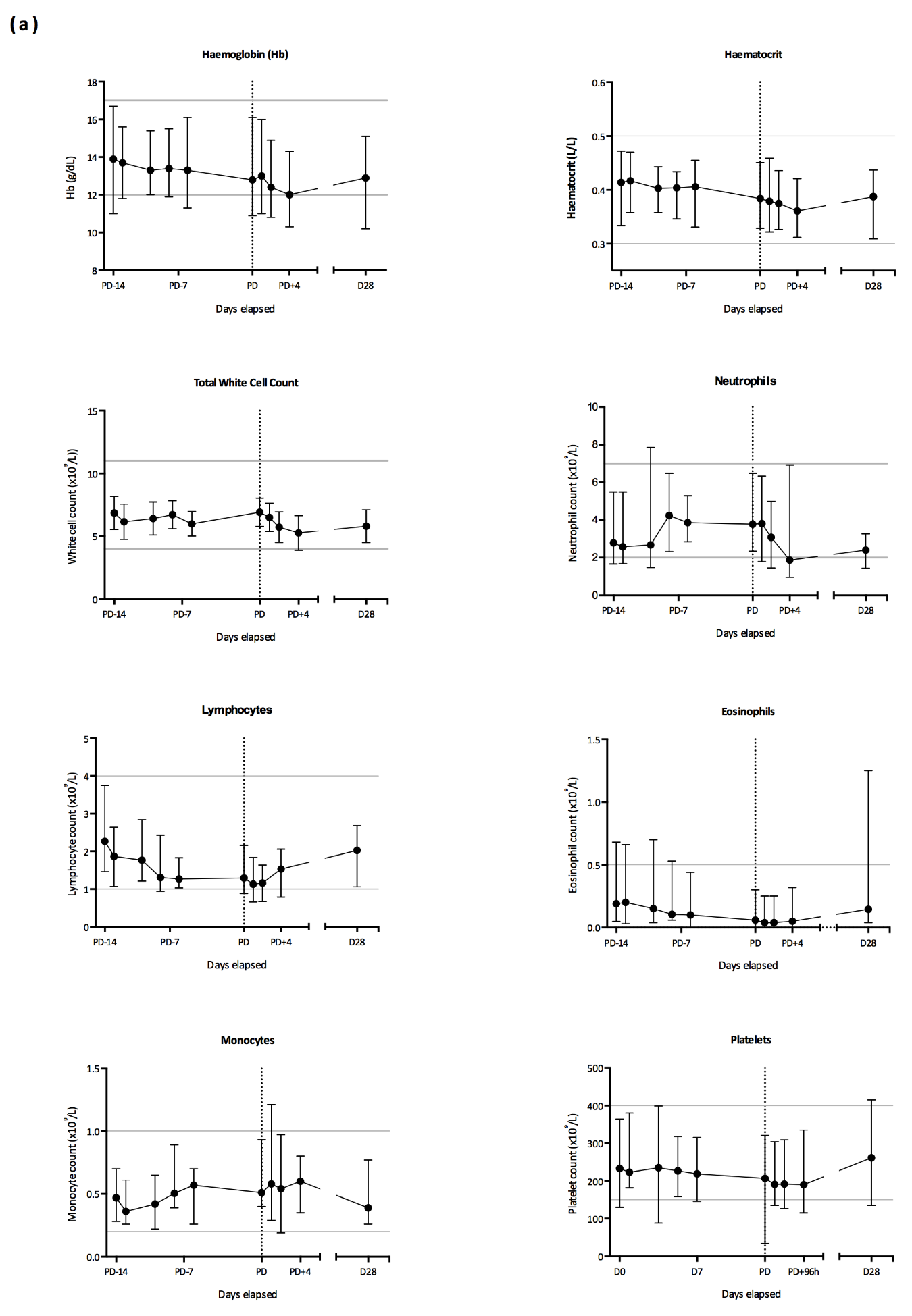


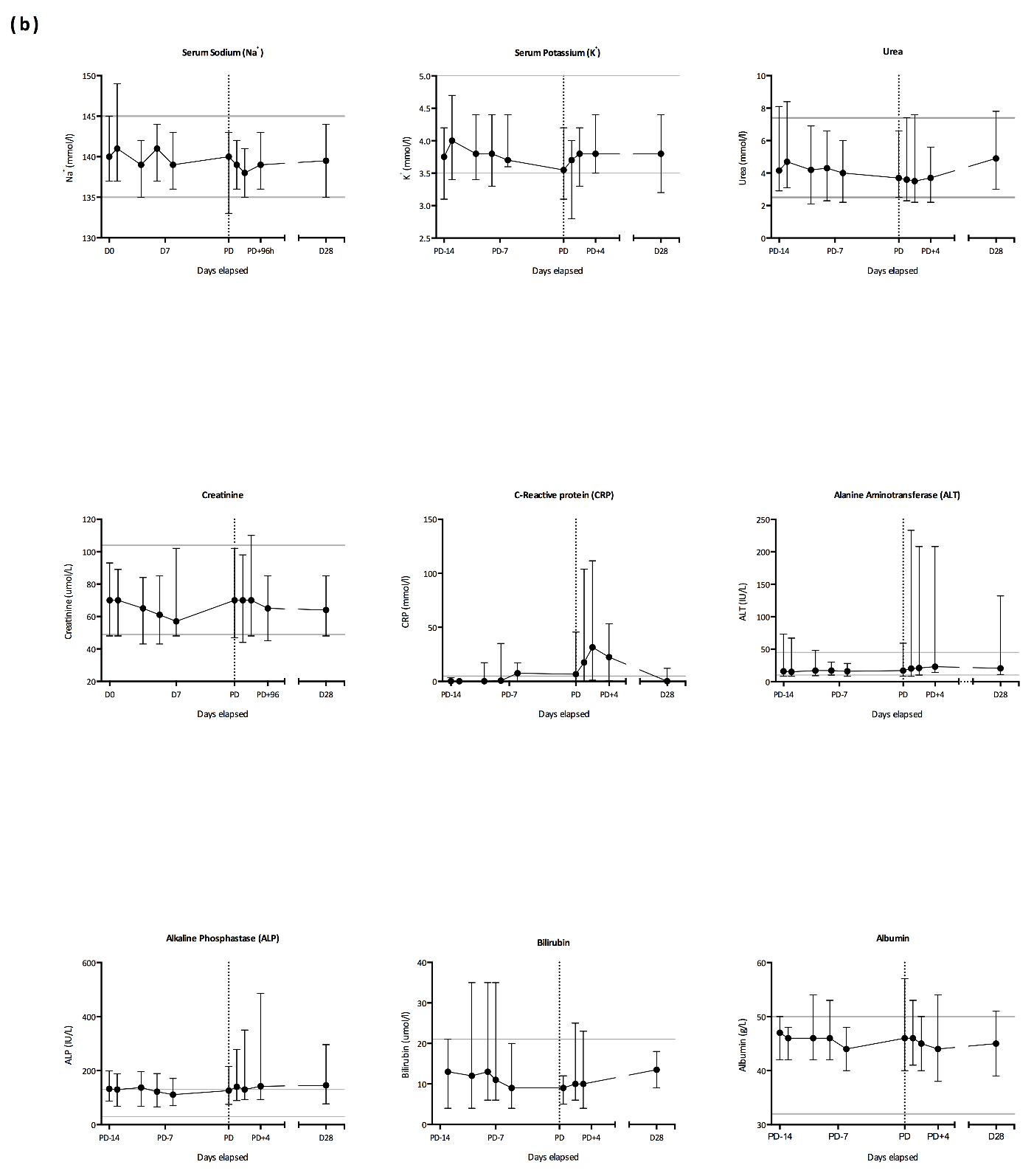


**Supplementary Figure 4**

IgA, IgG and IgM responses specific to **(a)** *S*. Paratyphi A O antigen and **(b)** *S*. Paratyphi flagellin (filled circles = paratyphoid diagnosis: n = 20; empty circles/dashed line = paratyphoid NOT diagnosed: n = 20). Plots indicate geometric mean concentrations, with error bars showing 95% confidence intervals. Asterisks represent p-values from linear regression models adjusted for dose and baseline values, comparing individuals meeting diagnostic criteria, with individuals NOT meeting diagnostic criteria. * p<0.05; **<0.01; *** p<0.001.

**
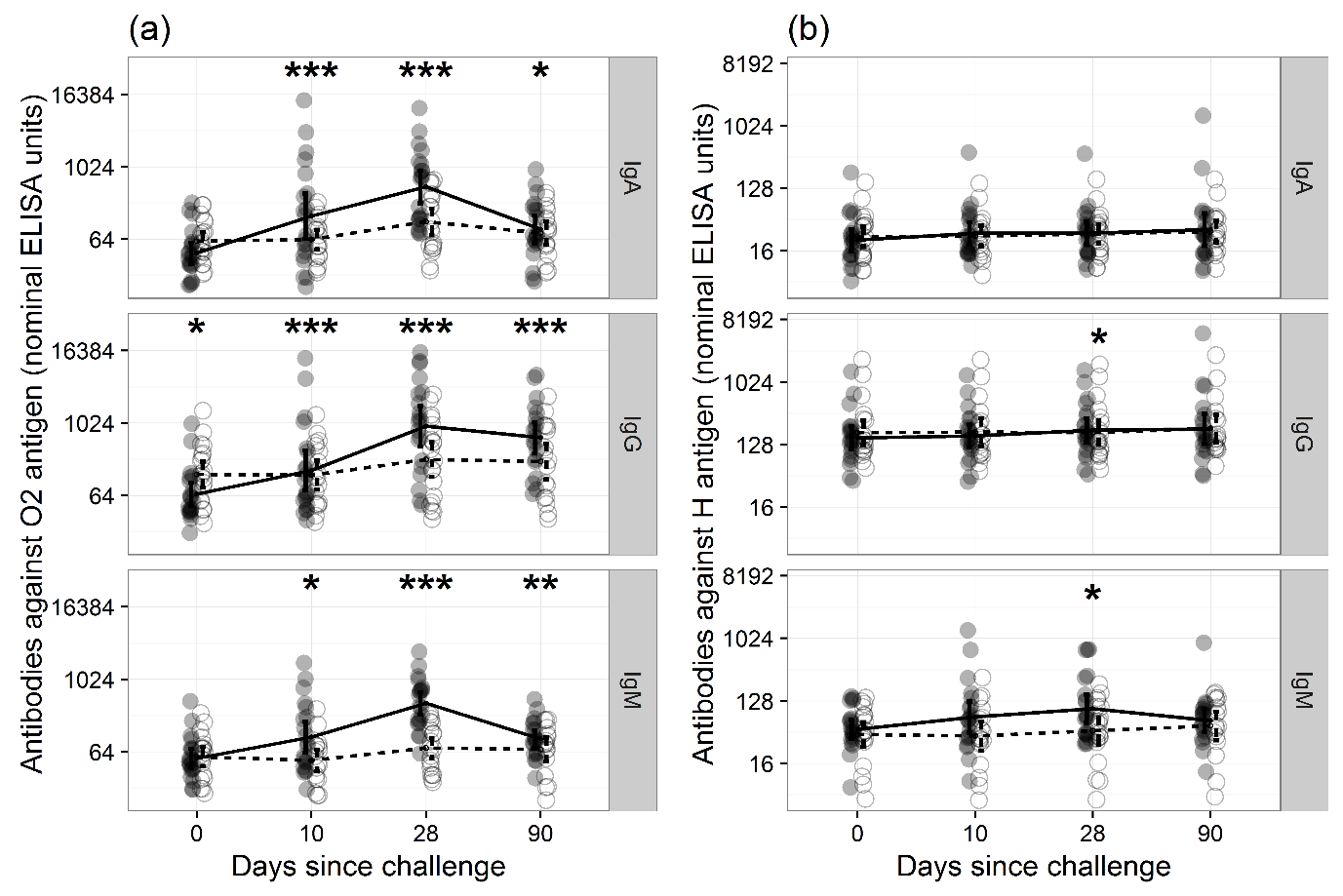
**

**Supplementary References**

1. Balasegaram S, Potter AL, Grynszpan D, et al. Guidelines for the public health management of typhoid and paratyphoid in England: practice guidelines from the National Typhoid and Paratyphoid Reference Group. J. Infect. **2012**; 65:197–213. Available at: http://www.sciencedirect.com/science/article/pii/S0163445312001491. Accessed 26 December 2015.

2. Li H, Handsaker B, Wysoker A, et al. The Sequence Alignment/Map format and SAMtools. Bioinformatics **2009**; 25:2078–9.

3. Zhou Z, McCann A, Weill FX, et al. Transient darwinian selection in salmonella enterica serovar paratyphi a during 450 years of global spread of enteric fever. Proc. Natl. Acad. Sci. U. S. A. **2014**; 111:12199–12204. Available at: http://www.scopus.com/inward/record.url?eid=2-s2.0-84906309033&partnerID=40&md5=5571050e865bae1e3bf0f1138318cbe7.

4. Micoli F, Rondini S, Gavini M, et al. A scalable method for O-antigen purification applied to various Salmonella serovars. Anal. Biochem. **2013**; 434:136–145. Available at: http://www.scopus.com/inward/record.url?eid=2-s2.0-84871989945&partnerID=40&md5=9bbb41863f50364330571512583e10a7.

5. Simon R, Curtis B, Deumic V, et al. A scalable method for biochemical purification of Salmonella flagellin. Protein Expr. Purif. **2014**; 102:1–7. Available at: http://dx.doi.org/10.1016/j.pep.2014.07.005.

6. Wayne, PA. Methods for dilution antimicrobial susceptibility tests for bacteria that grow aerobically; approved standard, 7th ed. Clinical and Laboratory Standards Institute, **2006.**
