# Supplementary figures and images for: Evaluation of the Clinical and Microbiological Response to Salmonella Paratyphi A Infection in the First Paratyphoid Human Challenge Model
Source: Clin Infect Dis. 2017 Feb 4;64(8):1066–73. doi: 10.1093/cid/cix042 (PMC5439345; doi:10.1093/cid/cix042)

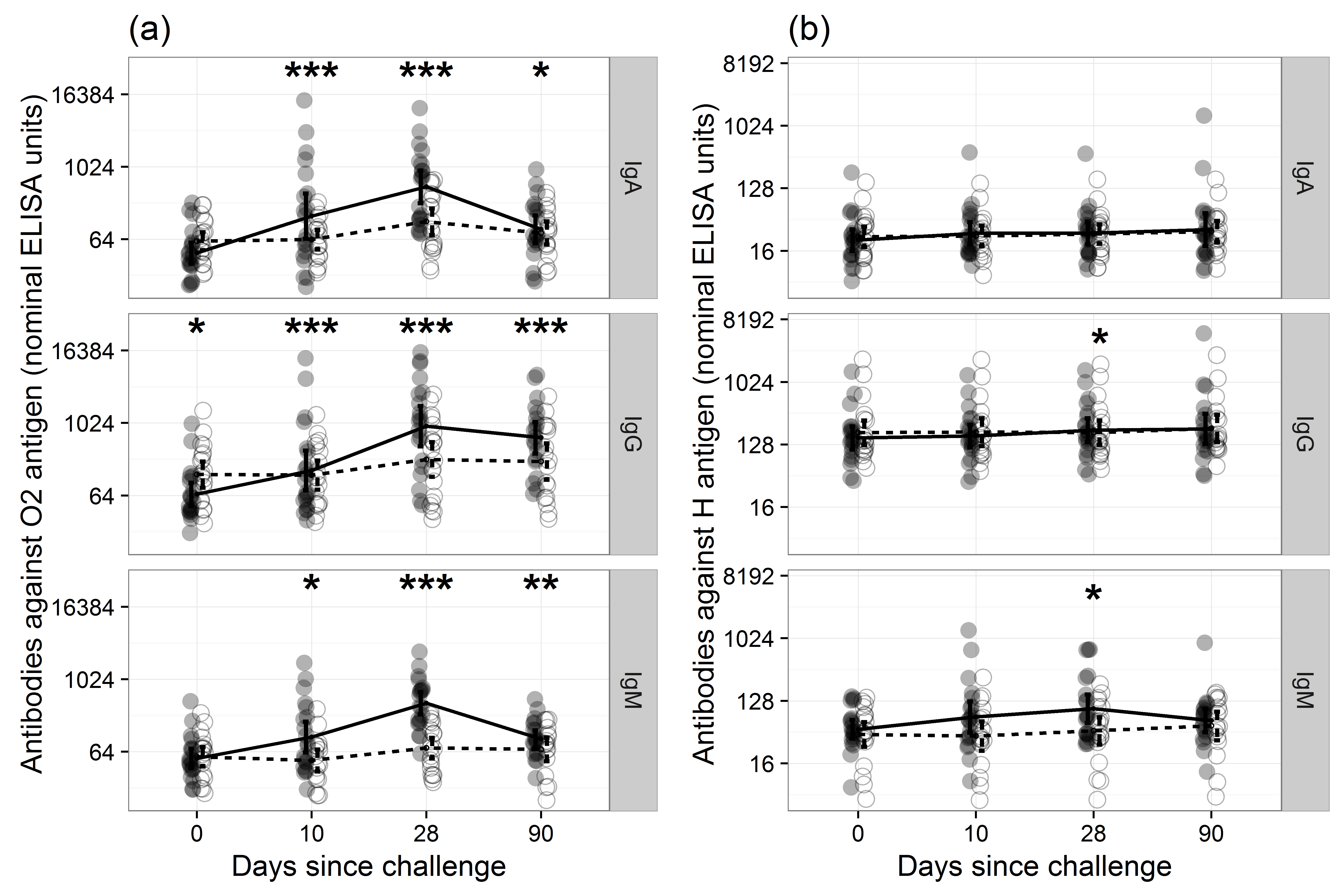

Supplement: Supplementary Data [file cix042_Supplementary_Data.zip › Supp_Fig4.TIFF]

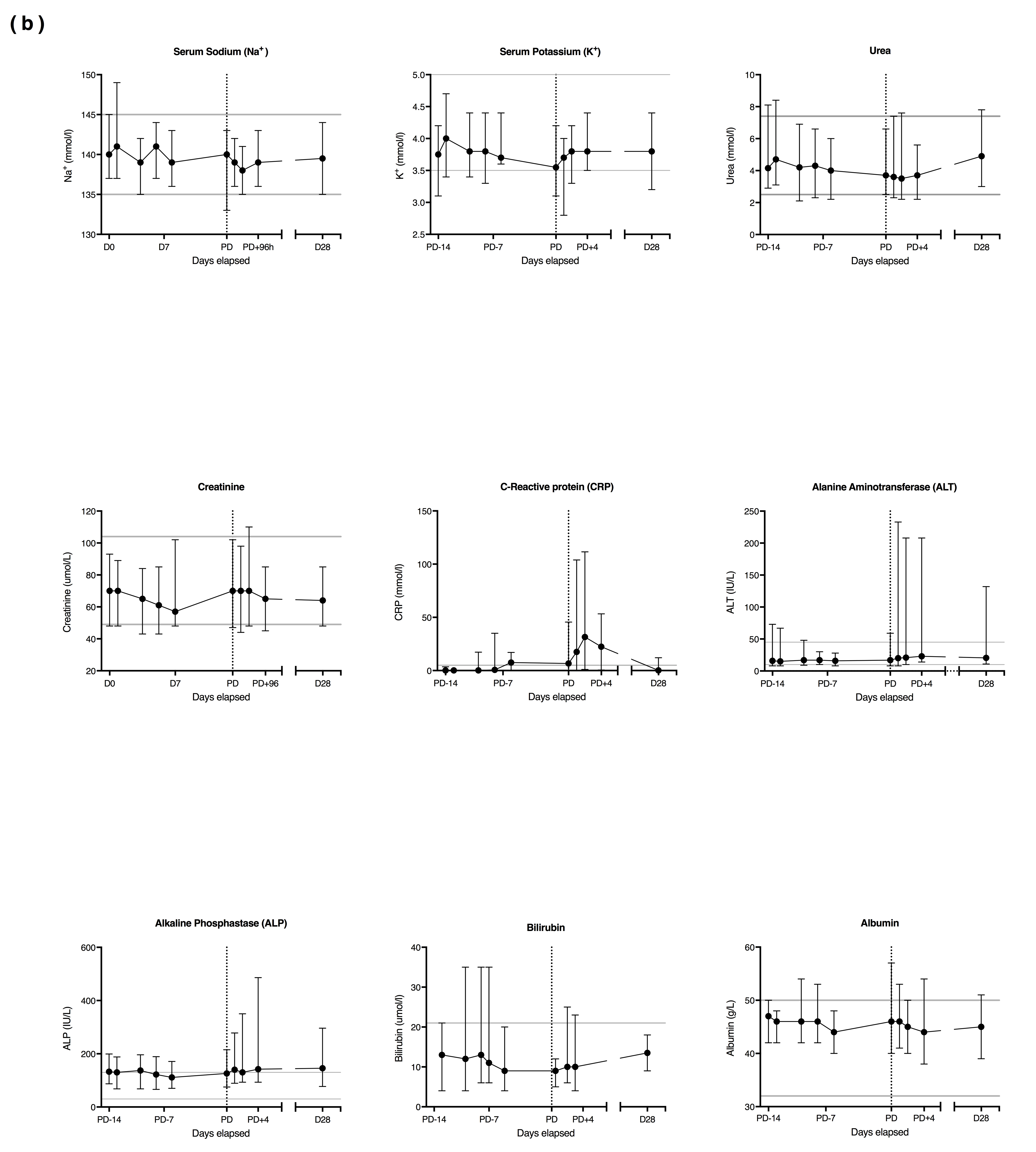

Supplement: Supplementary Data [file cix042_Supplementary_Data.zip › Supp_Fig3b.tiff]

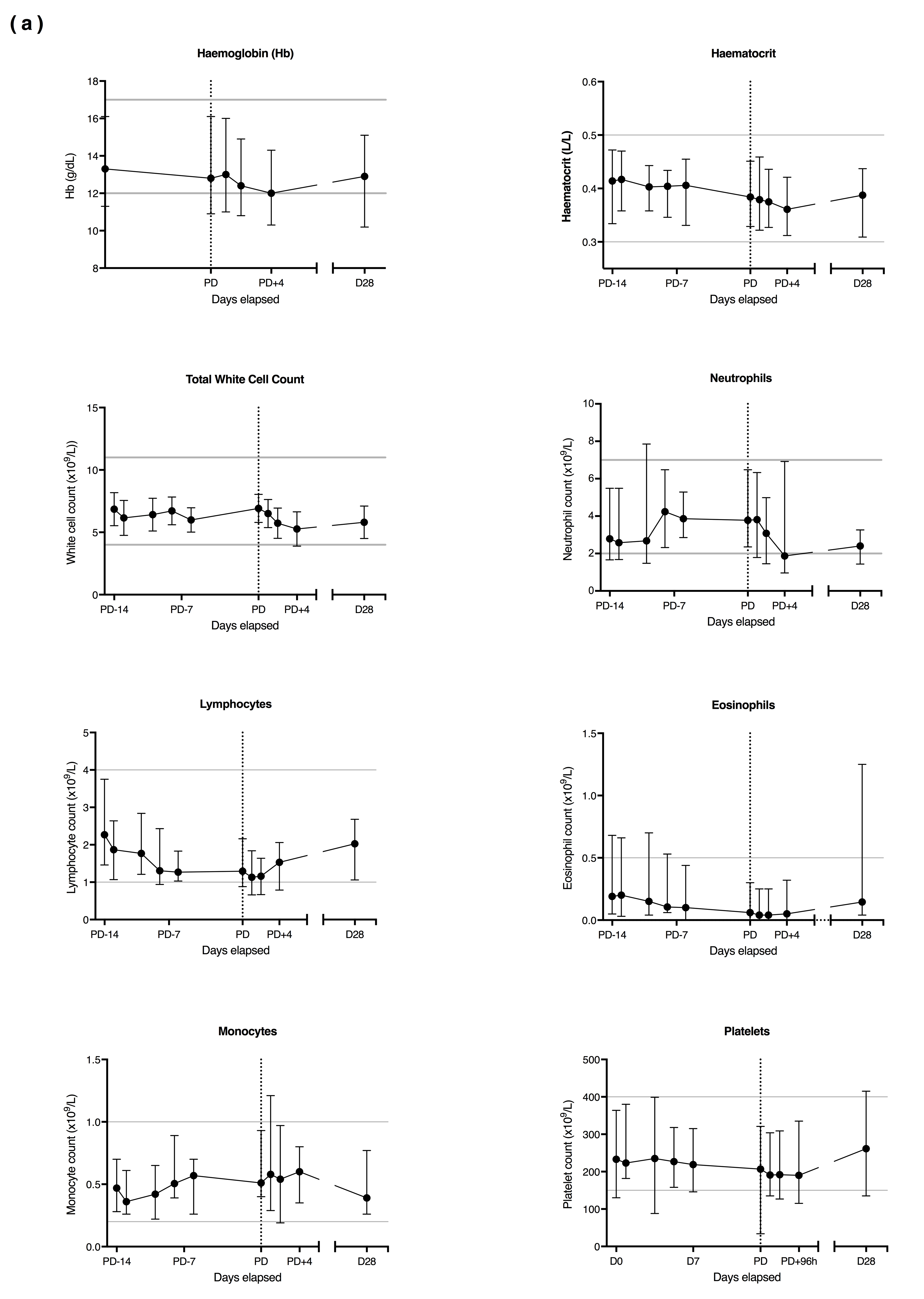

Supplement: Supplementary Data [file cix042_Supplementary_Data.zip › Supp_Fig3a.tiff]

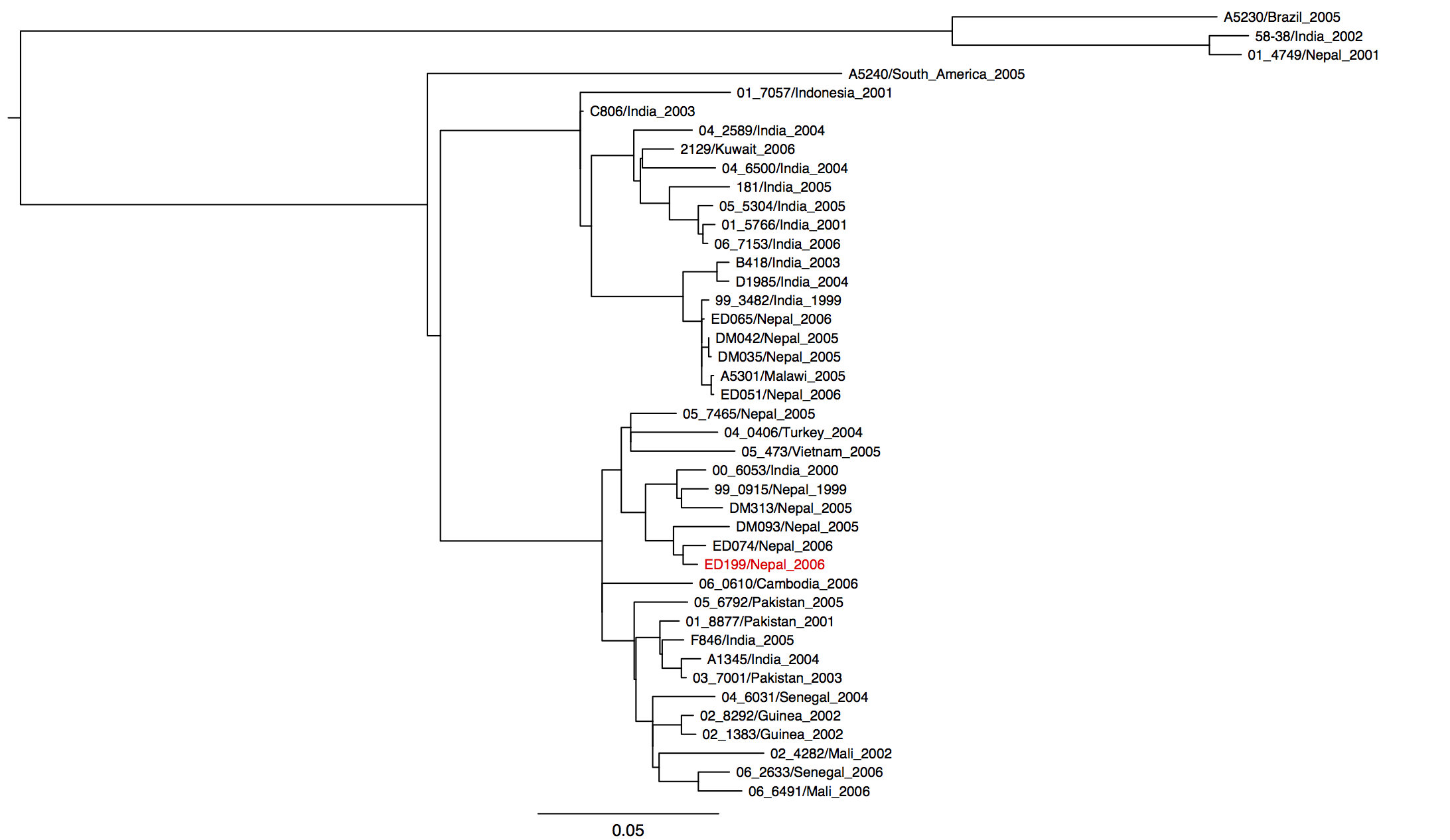

Supplement: Supplementary Data [file cix042_Supplementary_Data.zip › Supp_Fig_1.tiff]

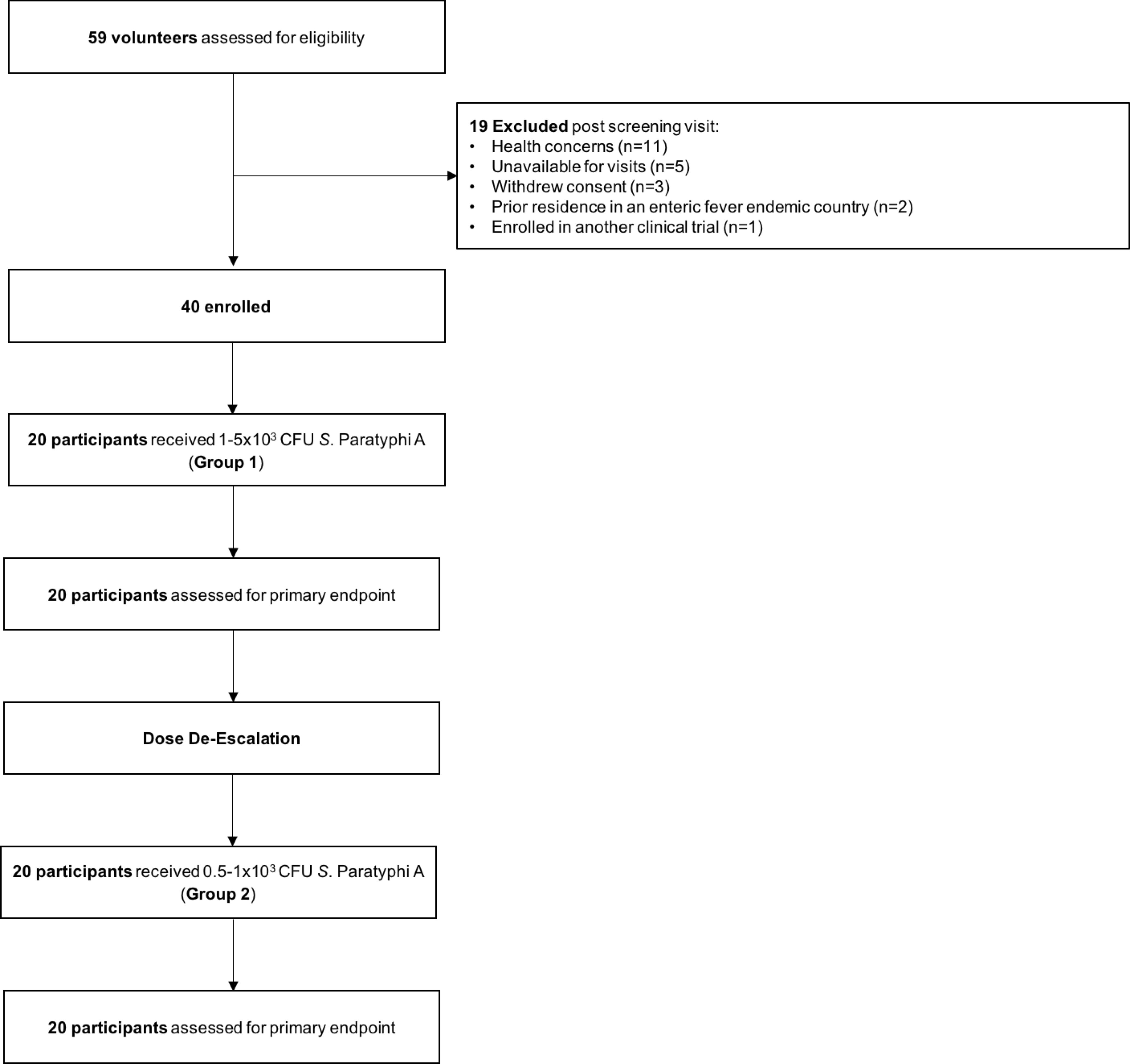

Supplement: Supplementary Data [file cix042_Supplementary_Data.zip › Supp_Fig_2.tiff]
